# The Alarmone Diadenosine Tetraphosphate as a Cosubstrate for Protein AMPylation

**DOI:** 10.1002/anie.202213279

**Published:** 2023-01-16

**Authors:** Matthias Frese, Philip Saumer, Yizhi Yuan, Doreen Herzog, Dorothea Höpfner, Aymelt Itzen, Andreas Marx

**Affiliations:** ^1^ Department of Chemistry Konstanz Research School Chemical Biology (KoRS-CB) University of Konstanz Universitätsstraße 10 78457 Konstanz Germany; ^2^ Institute of Biochemistry and Signal Transduction University Medical Center Hamburg-Eppendorf (UKE) Martinistraße 52 20246 Hamburg Germany; ^3^ Center for Structural Systems Biology (CSSB) University Medical Center Hamburg-Eppendorf Martinistraße 52 20246 Hamburg Germany

**Keywords:** AMPylation, Activity-Based Protein Profiling, Chemical Proteomics, Nucleotides, Post-Translational Modification

## Abstract

Diadenosine polyphosphates (Ap_*n*_As) are non‐canonical nucleotides whose cellular concentrations increase during stress and are therefore termed alarmones, signaling homeostatic imbalance. Their cellular role is poorly understood. In this work, we assessed Ap_*n*_As for their usage as cosubstrates for protein AMPylation, a post‐translational modification in which adenosine monophosphate (AMP) is transferred to proteins. In humans, AMPylation mediated by the AMPylator FICD with ATP as a cosubstrate is a response to ER stress. Herein, we demonstrate that Ap_4_A is proficiently consumed for AMPylation by FICD. By chemical proteomics using a new chemical probe, we identified new potential AMPylation targets. Interestingly, we found that AMPylation targets of FICD may differ depending on the nucleotide cosubstrate. These results may suggest that signaling at elevated Ap_4_A levels during cellular stress differs from when Ap_4_A is present at low concentrations, allowing response to extracellular cues.

## Graphical abstract

Ap_4_A was investigated for its potential applicability as a cosubstrate in AMPylation mediated by FICD, instead of the common cosubstrate ATP. Proficient AMPylation capability of Ap_4_A was observed, and new potential target proteins were found by chemical proteomics. Interestingly, AMPylation targets of FICD may differ depending on the cosubstrate.

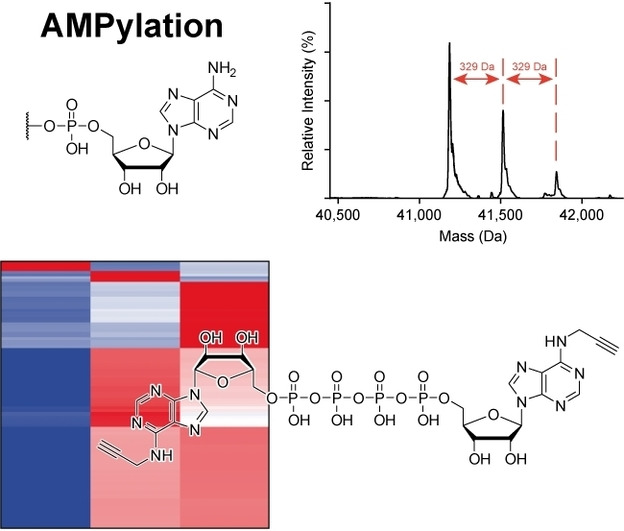

## Introduction

**Figure 1:**
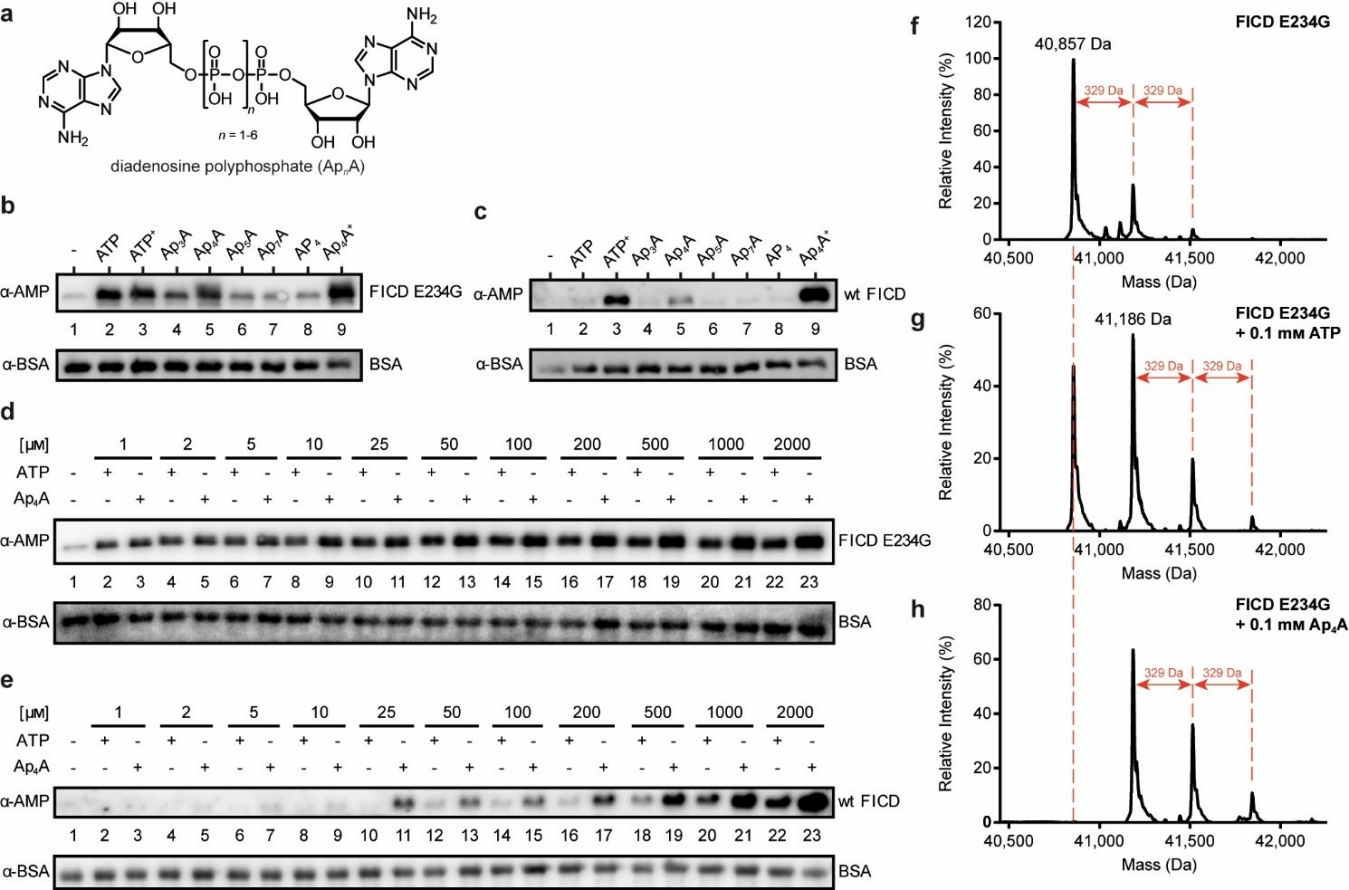
AutoAMPylation efficiency of FICD using different nucleotides serving as cosubstrates. a) Structure of Ap_*n*_As: two adenosines are connected at their 5′‐ends through a polyphosphate chain. The chain length of the symmetrical Ap_*n*_As can vary from 2 to 7. b) Impact of different nucleotides on autoAMPylation of FICD E234G (aa 102–458) was visualized by western blotting. The enzyme‐to‐nucleotide‐ratio was 1 : 200 (100 μM nucleotide), except for ATP^+^ and Ap_4_A^+^ with 2 mM concentration (1 : 4000). Detection of autoAMPylation was realized with an α‐AMP‐antibody, and BSA serving as loading control was detected with α‐BSA‐antibody. c) Same as (b) but with wt FICD (aa 102–458). d) Western blot analysis of the titration curve of ATP and Ap_4_A upon autoAMPylation reaction with FICD E234G. e) Same as (d) but with wt FICD. f) Intact protein LC–MS analysis of FICD E234G. The untreated protein is already monoAMPylated. g) Intact protein LC–MS analysis of FICD E234G incubated with ATP. The spectrum shows non‐AMPylated but also up to triple AMPylated protein upon ATP incubation. h) Same as (g), but the FICD E234G was incubated with Ap_4_A instead of ATP. Spectrum shows no residual non‐AMPylated protein.

Diadenosine polyphosphates (Ap_*n*_As) are symmetrical dinucleotides that were first discovered in biological systems in 1966.[^1^, ^2^] They consist of two adenosine moieties that are connected at their 5′‐ends through a polyphosphate chain (Figure 1a). The intracellular concentrations of Ap_*n*_As change in response to external stimuli (e.g., thermal and oxidative stress)[^3^, ^4^, ^5^, ^6^] and are therefore termed “alarmones” that signal cellular stress. The majority of Ap_*n*_A biosynthesis is believed to occur as a side reaction of aminoacyl‐tRNA synthetases.^[7]^ Moreover, it has been shown that Ap_4_A is formed as a side product in ubiquitin‐ and ubiquitin‐like‐activating enzymes.^[8]^

Despite the ubiquitous abundance of Ap_*n*_As across all kingdoms of life, only little is known about their cellular role. Thus far, their functions have been linked to apoptosis,^[9]^ cell proliferation,^[10]^ or DNA damage.^[11]^ Most recently, Ap_3_A in complex with the tumor suppressor protein Fhit was shown to hamper translation and thus reduce cell viability.^[12]^ Another study on the Ap_*n*_A interactome suggested that Ap_3_A and Ap_4_A are involved in metabolism and RNA‐related processes, amongst others.^[5]^

Since ATP and Ap_*n*_As share a high structural similarity, other biological processes are conceivable in which Ap_*n*_As may be used alongside ATP. In this context, the posttranslational modification (PTM) AMPylation caught our attention. AMPylation involves the enzymatic transfer of the adenosine monophosphate (AMP) moiety to Thr, Tyr or Ser side chains using ATP as a cosubstrate. The major writers of this PTM are the Fic (filamentation induced by cAMP) domain containing enzymes found across all kingdoms of life. While abundant in bacteria, eukaryotes generally encode one single Fic enzyme, known in humans as FICD.^[13]^ Localized in the ER lumen, FICD is capable of autoAMPylation in vitro and is also responsible for both AMPylation and deAMPylation of the HSP70 member BiP (HSPA5) in vivo linking this PTM to the unfolded protein response (UPR).[^14^, ^15^, ^16^, ^17^, ^18^, ^19^, ^20^, ^21^] Additionally, other studies demonstrate that metazoan Fic enzymes are involved in neuronal processes[^22^, ^23^, ^24^] and cytokine secretion.^[25]^ In 2019, the human pseudokinase SelO (and homologs) was identified as a new AMPylator being involved in redox homeostasis in mitochondria.^[26]^ In general, AMPylation appears to be involved in essential signaling pathways in eukaryotic cells.

In this study, we investigated Ap_*n*_As for their potential to serve as cosubstrates for AMPylation. We performed in vitro screening of several Ap_*n*_As for their use in the autoAMPylation reaction of FICD, where we identified Ap_4_A as viable candidate. The resulting autoAMPylation sites were investigated by tandem mass spectrometry (MS/MS) and were shown to be consistent with those previously reported for ATP.[^17^, ^18^] Our MS studies revealed that FICD shows higher autoAMPylation activity for Ap_4_A as the cosubstrate than for ATP.

Moreover, we identified 48 potential AMPylation target proteins from whole cell lysates that are specific for Ap_4_A as cosubstrate in an activity‐based protein profiling (ABPP) assay. These results were supported by a case study in which we found that the protein targets for AMPylation by FICD differed depending on whether ATP or Ap_4_A was used.

## Results and Discussion

To obtain the first insights into whether Ap_*n*_As are potential cosubstrates for AMPylation, we investigated autoAMPylation efficiency of FICD in vitro with a set of Ap_*n*_As in comparison to ATP. The (de)AMPylation activity of FICD is regulated by its capability to form homodimers: The monomeric form promotes AMPylation, whereas FICD dimers catalyze deAMPylation.[^16^, ^18^, ^27^, ^28^] In this context, Glu234 of FICD plays a crucial role in steering (de)AMPylation.[^14^, ^16^, ^27^, ^28^] In FICD dimers, Glu234 binds reversibly to Arg374 in the active site, impeding AMPylation‐competent ATP binding and restricting the enzyme to deAMPylation.[^27^, ^28^, ^29^, ^30^, ^31^, ^32^] Monomerization leads to an increase in Glu234 flexibility, disrupting this electrostatic bond and thus allowing AMPylation.^[28]^ The driving forces of the monomer‐dimer equilibrium of FICD remain elusive. However, recent findings suggest that the oligomeric state of FICD is modulated by ATP/ADP levels, where ATP increases the dimer dissociation rate and enhances monomerization of FICD.^[27]^ Since FICD wild‐type only shows detectable AMPylation activity at concentrations below the *K_d_* value of dimerization[^27^, ^28^] that are incompatible with many in vitro experiments, most reported results on FICD were historically obtained by employing deregulated FICD variants with mutated Glu234 (E234G) which—independent of oligomeric state of FICD—are unable to perform deAMPylation.

For this reason, we used two FICD constructs. The first tested construct was the deAMPylation‐deficient FICD fragment containing the E234G mutation, the second was “wild‐type” FICD (wt FICD, aa 102–458 for both constructs). Both variants were incubated either with ATP, Ap_3_A, Ap_4_A, Ap_5_A, Ap_7_A, or adenosine tetraphosphate (AP_4_) in a 1 : 200‐molar ratio (enzyme to nucleotide, 100 μM cosubstrate). Additionally, ATP and Ap_4_A were applied in a higher concentration (2 mM, henceforth termed ATP^+^ and Ap_4_A^+^, respectively). AP_4_ was included to monitor a possible influence of the phosphate chain length on AMPylation. Analysis of the experiments was performed by western blotting (WB) using a specific α‐AMP‐antibody.^[33]^ Besides ATP, Ap_3_A and Ap_4_A caused autoAMPylation of FICD E234G with Ap_4_A showing superior conversion to Ap_3_A (Figure 1b). At higher concentrations, incubation with Ap_4_A^+^ gave increased amounts of autoAMPylation relative to ATP^+^.

Since the wt FICD was employed at concentrations above the *K_d_* value of dimerization^[27]^ and should therefore predominantly be dimeric and AMPylation impaired, WB signal intensities were weaker. Nonetheless, autoAMPylation, especially for Ap_4_A, was still detectable owing to the high sensitivity of the α‐AMP‐antibody (Figure 1c). Interestingly, ATP^+^ and Ap_4_A^+^ led to increased autoAMPylation, supporting previous results that ATP enhances monomerization^[27]^ and giving rise to the assumption that Ap_4_A^+^ might act similarly. This observation prompted us to investigate a potential nucleotide concentration dependency for the autoAMPylation of FICD. For FICD E234G, autoAMPylation was already observable at lowest concentrations (1 : 2‐molar ratio, 1 μM nucleotide) but with increasing amounts of cosubstrate, autoAMPylation efficiency increased for Ap_4_A (Figure 1d). On the other hand, noticeable autoAMPylation of wt FICD was only detected for Ap_4_A starting from 25 μM while ATP displayed constantly less autoAMPylation than Ap_4_A (Figure 1e).

Next, we examined FICD autoAMPylation sites by peptide MS/MS to find out if different cosubstrates change the sites of automodification. For this, FICD E234G was incubated with either ATP or Ap_4_A in a 1 : 100‐molar ratio in vitro (enzyme to nucleotide, 100 μM cosubstrate). Regardless of the nucleotide, the same modification sites were identified for FICD E234G. These are Thr168 and Thr183 for both ATP and Ap_4_A as previously reported (Figure S6&S7, respectively).[^17^, ^18^] Interestingly, we determined Thr453 as another AMPylation site (Figure S8). Since both compounds are used for the same autoAMPylation sites, we measured intact protein liquid chromatography‐mass spectrometry (LC–MS) of FICD E234G to monitor a possible difference in conversion quantity. The purified recombinant protein was already monoAMPylated up to 37 % (Figure 1f). The addition of ATP as cosubstrate led to a strong monoAMPylation signal, but also a double and weak triple AMPylation signal were observed (Figure 1g). Ap_4_A instead resulted in a full conversion of the non‐AMPylated protein to a monoAMPylated and elevated double and triple AMPylated species (Figure 1h). To see if complete conversion is monitored with higher concentrations of ATP, the experiment was repeated with 1 mM ATP but the AMPylation pattern was similar to that for 100 μM (Figure S1). Therefore, we conclude that Ap_4_A is more efficiently used for FICD‐autoAMPylation than ATP.

AutoAMPylation of wt FICD was also monitored by MS. However, owing to the previously mentioned enzyme concentration above the *K_d_* value for dimerization, little AMPylation was detectable (data not shown). As for peptide MS/MS, the known AMPylation sites were identified, but with fewer peptide spectrum matches for each of the modified peptides than for FICD E234G. Intact protein MS yielded only non‐AMPylated protein even when using high nucleotide concentrations. On that basis, the western blot results using wt FICD (Figure 1c and e) were only possible due to the excellent sensitivity of the α‐AMP‐antibody. Nonetheless, concluding from the MS results of FICD E234G, Ap_4_A appears to be a viable cosubstrate for AMPylation.

**Figure 2:**
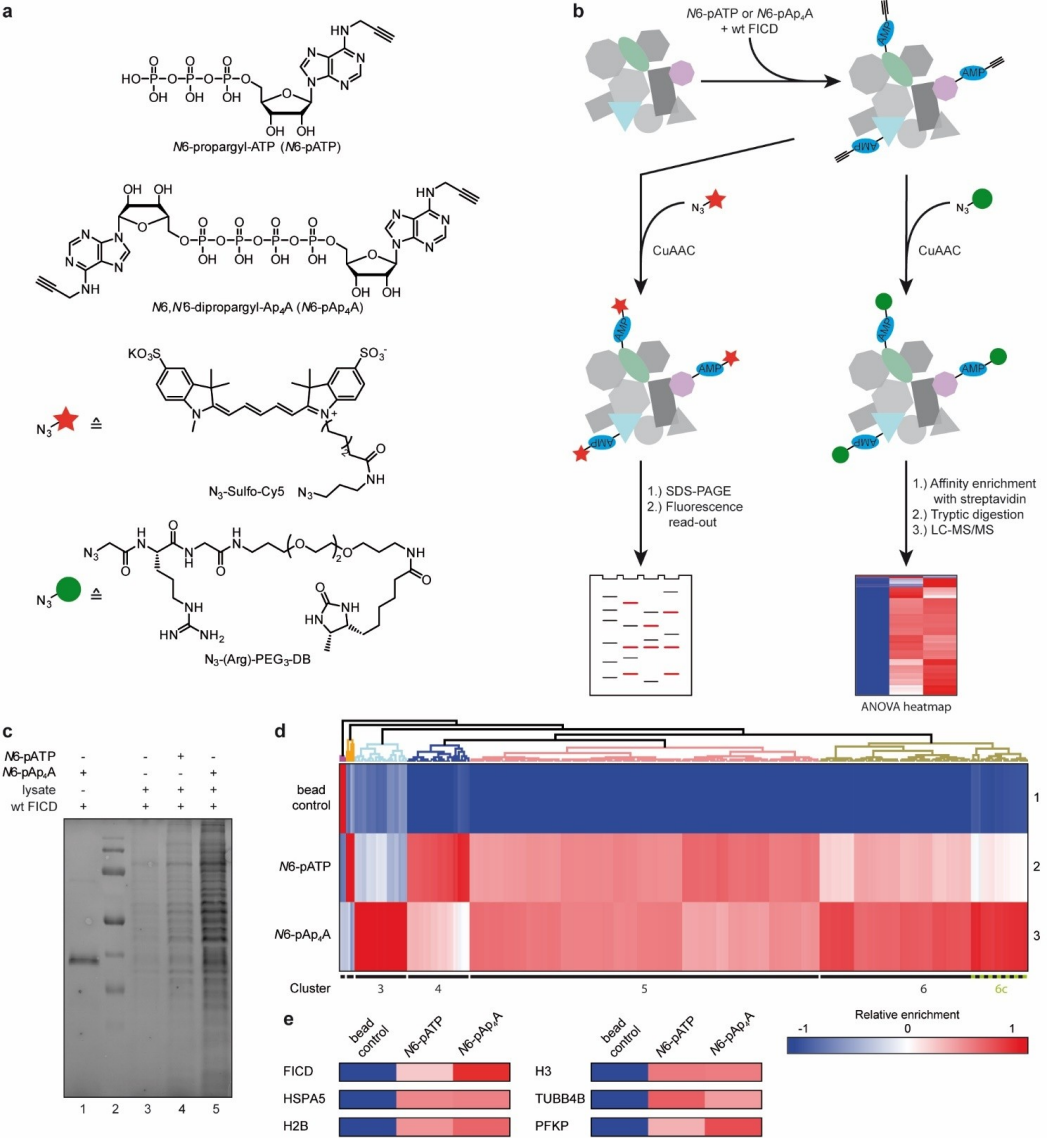
Identification of new AMPylation targets mediated by FICD using Ap_4_A as cosubstrate by ABPP. a) Chemical structure of compounds used for AP‐MS. *N*6‐propargyl‐ATP (top) and *N*6‐propargyl‐Ap_4_A (second structure) serving as chemical reporters. Third structure: N_3_‐Sulfo‐Cy5 used for CuAAC for fluorescence read‐out analysis; bottom: N_3_‐(Arg)‐PEG_3_‐DB used for affinity purification on Streptavidin‐immobilized agarose beads upon CuAAC. b) Workflow for the identification of new AMPylation targets. Left route: Incubation of the AMPylated cell lysates with N_3_‐Sulfo‐Cy5 to perform fluorescence read‐out. Right route: Incubation of the AMPylated cell lysates with N_3_‐(Arg)‐PEG_3_‐DB to perform affinity purification with Streptavidin‐immobilized agarose followed by LC–MS/MS. c) Fluorescence read‐out of HeLa KO lysate incubated with chemical reporters and wt FICD. d) Heatmap representation (*Z*‐scores) of enriched proteins that were found in at least 4 out of 6 replicates and passed the ANOVA‐based multiple‐sample test for statistically significant enrichment (FDR≤0.02, S_0_=0.3). The hits were hierarchically clustered by Euclidean distance. e) Excerpt of the ANOVA heatmap (d) of hits that were previously reported as potential AMPylation targets.

To date, several publications report insights into the AMPylome mediated by FICD and with ATP (or synthetic derivatives thereof) as cosubstrate.[^17^, ^22^, ^32^] Based on our finding that Ap_4_A can be used as cosubstrate by FICD in vitro, we aimed to find Ap_4_A‐specific AMPylation targets by ABPP from whole cell lysates employing a chemical reporter. A chemical handle on ATP which proved to be compatible with AMPylation reactions is a propargyl moiety on the adenosine's *N*6‐position.[^17^, ^22^, ^34^] After AMPylation, the alkyne moiety can react in a bioorthogonal copper(I)‐catalyzed azide‐alkyne cycloaddition (CuAAC) with azide conjugates bearing fluorophores or affinity tags to label AMPylated proteins for fluorescence read‐out or enrichment of AMPylated proteins, respectively. Hence, we designed and synthesized *N*6,*N*′6‐dipropargyl‐Ap_4_A (*N*6‐pAp_4_A) to serve as chemical reporter besides *N*6‐pATP (Figure 2a). As a model cell‐line, we chose *NUDT2*‐deficient HeLa cells (HeLa KO). NudT2 is an Ap_4_A‐specific hydrolase which degrades Ap_4_A to AMP and ATP.^[35]^ The hydrolase deficiency ensures that *N*6‐pAp_4_A will be less degraded before it is consumed for AMPylation.

We decided to monitor first if *N*6‐pAp_4_A resulted in any fluorescence signal upon AMPylation and CuAAC with a dye in cell lysates (Figure 2b, left route). For this, we incubated HeLa KO cell lysates with either *N*6‐pATP or *N*6‐pAp_4_A and spiked in wt FICD. Then, CuAAC with N_3_‐Sulfo‐Cy5 was performed to obtain dye‐labeled proteins followed by SDS‐PAGE and fluorescence read‐out. Additionally, we supplemented wt FICD with *N*6‐pAp_4_A to achieve autoAMPylation serving as positive control for CuAAC. Indeed, autoAMPylation of wt FICD was observed (Figure 2c, lane 1). As for the cell lysate, *N*6‐pAp_4_A (lane 5) produced an increased AMPylation signal than *N*6‐pATP (lane 4) and displayed additional bands compared to *N*6‐pATP (for comparison see Figure S2). Of note, experiments without wt FICD did not yield the same effect as with wt FICD underlining the specificity of the chemical reporter (Figure S3).

This result encouraged us to proceed with the affinity purification (Figure 2a, right route). HeLa KO cell lysate spiked with wt FICD was incubated with both chemical reporters but CuAAC was performed with an azide‐linker.^[36]^ The linker consists of an azide and a desthiobiotin (DB) tag for affinity purification flanking an arginine and a PEG_3_ chain (N_3_‐(Arg)‐PEG_3_‐DB, Figure 2b).^[36]^ Purification of AMPylated proteins was realized through binding via the DB tag to streptavidin agarose beads. DB was used instead of biotin as it still exhibits sufficient binding affinity to streptavidin and the elution conditions do not impair in‐solution tryptic digestion before LC–MS/MS measurements.[^37^, ^38^, ^39^] During digestion, the arginine enables cleavage of the bulky PEG_3_‐DB moiety facilitating later ionization of the AMPylated peptides. The affinity enrichment was performed in biological triplicates and the LC–MS/MS analysis was conducted in technical duplicates resulting in a total of 6 measurements per condition. Identification and label‐free quantification (LFQ) of enriched proteins were performed with MaxQuant.[^40^, ^41^] A total of 1317 proteins were identified with MaxQuant. Further statistical analysis was performed with Perseus.^[42]^ Protein hits that were found in at least 4 out of 6 replicates of one condition (bead control, *N*6‐pATP or *N*6‐pAp_4_A) were deemed as valid leaving 582 proteins. For these, missing values were imputed from a normal distribution (width 0.3, downshift 1.8) for each replicate separately. To identify significantly enriched proteins for each of the chemical reporters, a one‐way ANOVA multiple‐sample test (FDR≤0.02, S_0_=0.3) was performed. As a result, 298 significantly enriched proteins were identified for being potentially AMPylated by ATP or Ap_4_A (Supporting Information Data 1). The hits were hierarchically clustered and visualized in a heatmap (Figure 2d). The color scale represents the median *Z*‐score normalized LFQ as calculated by Perseus. As a result, 6 clusters were obtained. Cluster 4 showed a slight preference for *N*6‐pATP while clusters 5 and 6 comprised hits that displayed enrichment for both *N*6‐pATP and *N*6‐pAp_4_A. In these, we found FICD (cluster 6) and HSPA5 (cluster 5) but also other potential AMPylation targets which have been previously reported, i.e., histones H2B^[43]^ and H3,^[43]^ TUBB4B^[17]^ (all cluster 5), and PFKP^[22]^ (cluster 6, Figure 2e).

**Figure 3:**
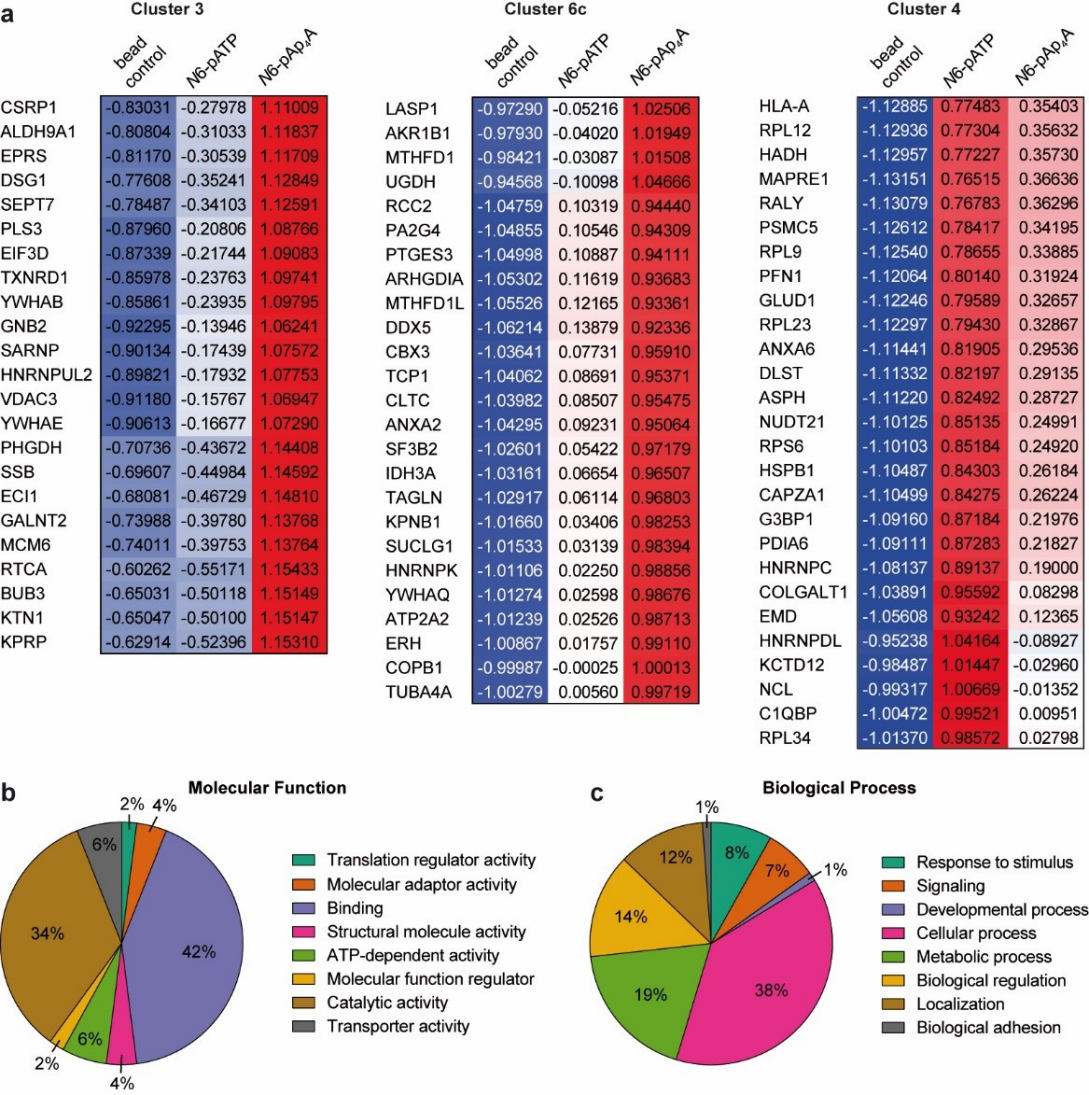
GO term analysis of Ap_4_A‐enriched protein hits. a) ANOVA heatmap excerpts of cluster 3 (23 hits) and cluster 6c (25 hits) depicting hits that show high enrichment for Ap_4_A. Cluster 4 displays hits with enrichment for ATP. Depicted values are the *Z*‐scores of the respective protein hit and condition. b) Molecular function analysis of the Ap_4_A‐enriched hits from clusters 3 and 6c with the PANTHER tool. c) Biological process analysis of the Ap_4_A‐enriched hits from clusters 3 and 6c with the PANTHER tool.

Remarkably, cluster 3 contained 23 proteins exclusively enriched for *N*6‐pAp_4_A (Figure 3a) of which only EIF3D was described earlier as potential AMPylation hit.^[44]^ Furthermore, cluster 6c (Figure 2d) displayed also strong enrichment for *N*6‐pAp_4_A compared to *N*6‐pATP containing 25 hits (Figure 3a). Overall, the *Z*‐score difference of *N*6‐pAp_4_A and *N*6‐pATP of these total 48 protein hits was ≥ 0.75. For further analysis of the 48 hits, we used functional classification with respect to their gene ontology (GO) terms in the PANTHER database (Supporting Information Data 2)^[45]^ and also examined GO annotation with the DAVID database (Supporting Information Data 3).[^46^, ^47^] Regarding their biological process annotation, the *N*6‐pAp_4_A‐enriched hits are involved in various pathways (Figure 3c). A major portion takes part in cellular processes while roughly one fifth is involved in metabolism, too. The connection to signaling and stimulus responses is also of note since Ap_*n*_As and AMPylation are linked to these processes. As for the molecular function annotation, 42 % of hits are linked to binding (Figure 3b). This binding can be largely split into nucleotide, RNA and protein binding (Supporting Information Data 3). The location of numerous hits according to cellular components are in the cytoplasm or cytosol and also have association with the membrane or diverse organelles such as the nucleus, ER and mitochondrion (Supporting Information Data 2 and 3). Considering the enrichment significance in terms of the *p*‐value (also adjusted using Bonferroni or Benjamini correction), no noteworthy enrichment was determined, as *p*‐values were rather high (Supporting Information Data 3). This indicates that AMPylation of Ap_4_A is involved in numerous processes.

**Figure 4:**
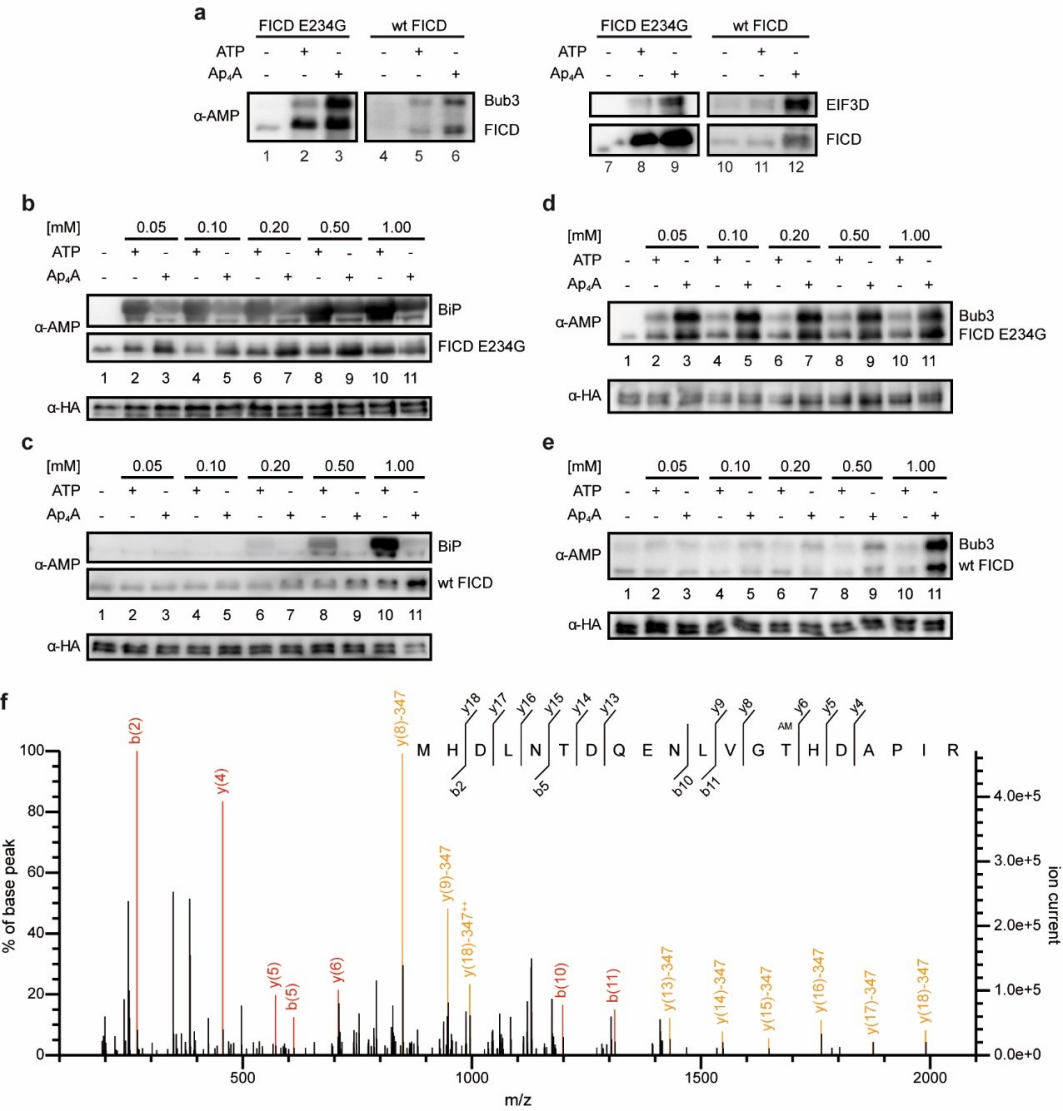
Target AMPylation of BiP and newly identified Bub3 mediated by FICD. a) Western blot of two representative proteins from the ABPP which are Bub3 and EIF3D. Both proteins were checked on their AMPylation potential by FICD E234G and wt FICD. b) Western blot of the concentration dependent AMPylation of BiP mediated by FICD E234G using ATP or Ap_4_A as cosubstrate. Detection of AMPylation was realized with an α‐AMP‐antibody and HA serving as loading control against α‐HA‐antibody. c) Same as (a) but with wt FICD. d) Similar to (b) but instead of BiP, Bub3 was used for target AMPylation mediated by FICD E234G. e) Same as (d) but with wt FICD. f) MS/MS spectrum of AMPylated peptide (single peptide ID) containing Thr94 of Bub3 using Ap_4_A as cosubstrate. MS/MS spectrum for the peptide was selected based on the highest score (−10lgP) assigned by Mascot. Fragment ions with a characteristic AMPylation associated neutral loss are shown in yellow.

Based on the outcome of the ABPP, we checked the mitotic checkpoint protein BUB3 (Bub3) and EIF3D representatively for their AMPylation potential by FICD E234G and wt FICD. Indeed, both proteins were AMPylated by both FICD constructs using preferably Ap_4_A as cosubstrate (Figure 4a). We then investigated BiP and Bub3 in further detail. In ABPP, BiP was found in cluster 5 (HSPA5) and served as reference since it is the most prominent AMPylation target of FICD. BiP is a key chaperone in the UPR.^[19]^ During ER homeostasis, the chaperone‐activity of BiP is inhibited due to AMPylation. Upon ER stress, FICD deAMPylates BiP allowing the chaperone to take over its function in the UPR.[^14^, ^15^, ^20^, ^21^]

We first tested FICD E234G for the AMPylation of BiP with ATP and Ap_4_A as cosubstrates in different concentrations in vitro. Since BiP possesses ATP hydrolysis activity, we used the hydrolysis‐deficient T229A construct (aa 19–654).[^20^, ^48^, ^49^] AMPylation was monitored by western blotting with the anti‐AMP‐antibody. The AMPylation efficiency of BiP was apparently stronger for ATP than for Ap_4_A independent of the nucleotide concentration (Figure 4b). Using wt FICD, AMPylation was only observed for higher ATP concentrations but not for Ap_4_A (Figure 4c). For a successful AMPylation reaction BiP must inhabit its ATP‐bound state.^[21]^ To check if Ap_4_A could be used as cosubstrate for AMPylation but is unable to mimic/induce the ATP‐bound state of BiP, we repeated the experiments by addition of non‐hydrolyzable ATP (ApCpp) to natural Ap_4_A (Figure S4). However, no difference was observed in comparison to the experiments without ApCpp. To ensure specificity of target AMPylation, FICD E234G was incubated with BSA which is not known to be AMPylated and indeed no significant AMPylation was detected (Figure S5). Furthermore, the AMPylation site was identified by peptide MS/MS to be Thr518, as has been previously reported (Figure S9).[^18^, ^20^]

As for Bub3, it is one of the 48 identified protein hits that were highly enriched for *N*6‐pAp_4_A. Bub3 is crucial for the proper establishment of kinetochore‐microtubule attachments during metaphase.^[50]^ In line with the ABPP data, AMPylation efficiency of Bub3 after incubation with FICD E234G and with Ap_4_A as cosubstrate was found to be more efficient than for ATP (Figure 4d). A similar result was observed for wt FICD where only high Ap_4_A concentrations led to a detectable AMPylation (Figure 4e). By peptide MS/MS, we identified three Bub3 AMPylation sites Thr94 (Figure 4f and Figure S10), Thr133 and Thr142 (Figure S11&S12, respectively). Thr94 and Thr133 were AMPylated using either cosubstrate but at least twice as many AMPylated peptide spectrum matches were found for Ap_4_A than for ATP with the same amount of protein input. Interestingly, we found AMPylated peptides of Thr142 only for Ap_4_A.

## Conclusion

Recently, FICD was linked to neurogenesis^[22]^ and several in vitro targets were identified,[^17^, ^22^, ^43^, ^44^, ^51^] but the only well understood in vivo target remains BiP which puts AMPylation into a stress response‐related context. ATP is inarguably the cosubstrate for this PTM. However, Ap_*n*_As are increasingly formed in response to stress[^3^, ^4^, ^5^, ^6^] (“alarmones”) and structurally similar to ATP. Hence, we investigated if Ap_*n*_As are potential cosubstrates for AMPylation. Here, we show that some but not all investigated dinucleoside polyphosphates are cosubstrates for autoAMPylation of FICD in vitro (Figure 1b, c). Interestingly, FICD uses Ap_4_A more efficiently for autoAMPylation than ATP. In addition to that, MS analysis of FICD E234G revealed that Ap_4_A led to a full conversion of the enzyme to its AMPylated forms (Figure 1h) while the usage of ATP resulted in residual non‐AMPylated FICD (Figure 1g). However, consumption of Ap_4_A as cosubstrate in AMPylation potentially generates ATP as product doubling the nominal concentration of cosubstrate.

In addition, Ap_4_A might serve as precursor or surrogate of ATP in general. As it was previously shown for the *H. somni* Fic enzyme IbpA, ATP can be hydrolyzed by Fic enzymes without the use in AMPylation.^[44]^ This side‐effect reduces the overall concentration of cosubstrate, simultaneously diminishing auto AMPylation efficiency. With its second adenosine, Ap_4_A represents a “capped” ATP, and might therefore be more stable towards hydrolysis. This nominal concentration effect in combination with reduced cosubstrate hydrolysis might play into the increased autoAMPylation efficiency of FICD. Nevertheless, higher concentrations of ATP resulted in no significantly increased autoAMPylation in full‐length MS analysis (Figure S1). Therefore, we conclude that Ap_4_A is indeed a better cosubstrate for autoAMPylation of FICD. Nevertheless, Ap_4_A might serve as a potential in vitro tool protecting ATP from hydrolysis.

Regarding the AMPylation sites of FICD, no difference was found regardless of the cosubstrate. Besides the published AMPylation sites (Thr168 and Thr183),[^17^, ^18^] we additionally identified a third site when either cosubstrate was used (Thr453).

Previous reports already have suggested that autoAMPylation has an effect on the enzyme's overall activity.^[18]^ Furthermore, it was suggested that ATP/ADP levels have impact on the oligomeric state of FICD. A shift to the ATP side leads to monomerization and thus to increased AMPylation activity.^[27]^ Using wt FICD, we observed autoAMPylation with Ap_4_A as cosubstrate in lower concentrations than ATP and generally more efficiently (Figure 1d and e). This could indicate that Ap_4_A also has influence on the oligomeric state. Ap_4_A concentrations in mammalians range between 0.05 and 1 μM under non‐stressed conditions^[52]^ while stress stimuli in various organisms were reported to increase these levels 2‐ to 100‐fold.[^3^, ^4^, ^5^, ^6^] However, Ap_4_A accumulation in cellular compartments is unknown. Competitive binding of Ap_4_A and ATP was reported for histidine triad nucleotide‐binding protein 1^[53]^ and also in the purinergic receptor system.^[54]^ Therefore, it is conceivable that Ap_4_A might get involved in the ATP/ADP balance pushing FICD to the monomer side leading to enhanced AMPylation and consequently reduced deAMPylation activity.

Since the only known AMPylation targets are modified with ATP as a cosubstrate, we searched for new AMPylation targets mediated by FICD including Ap_4_A. Based on earlier publications using *N*6‐modified adenosine compounds as feasible chemical reporters,[^17^, ^22^, ^34^] we designed and synthesized *N*6‐pAp_4_A for ABPP. Our studies using HeLa KO cell lysates with wt FICD resulted in a total of 298 protein hits that are potential targets for AMPylation either by ATP or Ap_4_A (Figure 2d). Among these hits we identified FICD's main substrate BiP and other previously reported in vitro targets[^22^, ^43^, ^44^, ^51^] supporting the validity of our ABPP and strengthening earlier reports.[^17^, ^22^] However, 48 protein hits (cluster 3 and 6c, Figure 2d, Figure 3a) were highly enriched for *N*6‐pAp_4_A pointing to the notion that Ap_4_A modulates AMPylation towards other targets than ATP.

In a case study, we herein demonstrate for AMPylation of BiP, that ATP is the more proficient cosubstrate, while Ap_4_A is the more proficient cosubstrate for Bub3 (Figure 4). The most likely explanation for Ap_4_A being a poor cosubstrate for BiP may lie in the protein's mode of action. For a successful AMPylation, BiP must bind ATP in its active center leading to allosteric alteration of the protein.^[21]^ Thus, the AMPylation site (Thr518) will be presented and FICD can perform the catalysis of the PTM.^[21]^ Conclusively, BiP AMPylation requires two ATP molecules: one for allosteric binding, one for PTM formation. In the case of Ap_4_A, BiP might not initiate the conformational change leaving the Thr518 blocked for AMPylation. However, the addition of ApCpp serving as ATP surrogate for BiP binding did not change AMPylation efficiency using Ap_4_A as cosubstrate (Figure S4). The situation is in turn different for the AMPylation of Bub3 since the efficiency was higher for Ap_4_A. To our comprehension, Ap_4_A is accordingly the preferred cosubstrate for FICD‐catalyzed Bub3 AMPylation. Additionally, one AMPylation site for Bub3 was identified which was modified exclusively by Ap_4_A serving as cosubstrate. The mechanistic origin for this cosubstrate specificity is currently unclear and requires additional comprehensive investigations.

Altogether, we report in this study that FICD‐catalyzed AMPylation targets may differ whether ATP or the alarmone Ap_4_A is employed as cosubstrate at least in vitro. This finding links for the first time AMPylation to Ap_4_A alarmone formation as another cellular stress response.

## Conflict of interest

The authors declare no conflict of interest.

## Supporting information

As a service to our authors and readers, this journal provides supporting information supplied by the authors. Such materials are peer reviewed and may be re‐organized for online delivery, but are not copy‐edited or typeset. Technical support issues arising from supporting information (other than missing files) should be addressed to the authors.

Supporting Information

Supporting Information

Supporting Information

Supporting Information
