# Supplementary material for: The Alarmone Diadenosine Tetraphosphate as a Cosubstrate for Protein AMPylation
Source: Angew Chem Int Ed Engl. 2023 Jan 16;62(8):e202213279. doi: 10.1002/anie.202213279 (PMC10107192; doi:10.1002/anie.202213279)
Supplement: Supplementary file 1 — Supporting Information [file ANIE-62-0-s002.pdf]

Angewandte Chemie

Eine Zeitschrift der Gesellschaft Deutscher Chemiker

Supporting Information

The Alarmone Diadenosine Tetraphosphate as a Cosubstrate for Protein AMPylation

*M. Frese, P. Saumer, Y. Yuan, D. Herzog, D. Höpfner, A. Itzen, A. Marx**

Table of Contents

Experimental Procedures 3
Protein expression and purification. 3
In vitro AMPylation assays. 3
Western blot. 4
Cell culture and lysis. 4
Fluorescence analysis. 4
Affinity purification assay. 4
LC-MS/MS measurement of affinity purification samples. 4
Data analysis and quantification of affinity purification assays. 5
In gel digestion and sample preparation. 5
LC-MS/MS measurement of in gel digested proteins. 5
Identification of AMPylated peptide fragments. 5
LC-MS measurement and analysis of full-length proteins. 6
MS data deposition 6
Chemical synthesis of of *N*6, *N* 6''-dipropargyldiadenosine-*O*5',*O*5'''-tetraphosphate (sodium salt form, 3) 6
Supporting Results 8
References 14
Author Contributions 14

Experimental Procedures

All solvents and reagents were commercially available and were used without further purification. The quality of the solvents was at least *pro analysi* or higher. For reactions in water or buffer solutions MilliQ® (MQ) water prepared by Merck Millipore BioPak® ultrapure water system was used. The reactions were performed using standard laboratory techniques. The reactions were generally performed under the exclusion of air and moisture. The solvents were dried over molecular sieve as needed. Nucleotides were purified by anion-exchange chromatography on an Äkta purifier (GE Healthcare) using a DEAE Sephadex™ A-25 (GE Healthcare Bio Sciences AB) column with a linear gradient of 0.0 M to 1.0 M triethylammonium bicarbonate buffer (TEAB, pH 7.5). Reversed phase high-pressure liquid chromatography (RP-HPLC) was performed using a Shimadzu Prominence system equipped with preparative LC-20AP pumps. Nucleotides were purified using a VP 250/21 NUCLEODUR C18 Htec, 5 µm (Macherey-Nagel) column with a linear gradient of 5% to 40% ACN in 50 mM TEAB and a flow rate of 8 mL/min. NMR spectra were recorded on an Avance III 400 MHz (Bruker) and Avance Neo 800 MHz (Bruker) spectrometer. ^{1}H, ^{13}C and ^{31}P chemical shifts are reported relative to the solvent peak and are given in ppm (δ); s: singlet, d: duplet, t: triplet, q: quartet, m: multiplet. HR-ESI-MS was measured with MassHunter (version 10.1.62, Agilent) on a 6546 QTOF (Agilent) system and evaluated with MassHunter Qualitative Analysis (version 10.0.10305.0, Agilent). The reported yield refers to the analytically pure substance and is not optimized.

Protein expression and purification.
For FICD constructs, His_6-Halo-TEV fusion proteins of wt FICD (aa 102-458) and FICD E234G (aa 102-458) coded on a pHAL vector were expressed in *E. coli* BL21-CodonPlus (DE3)-RIL in LB medium containing 100 µg/mL Carbenicillin and 34 µg/mL Chloramphenicol to a density of A600 = 0.6–0.7. Protein expression was induced at 23 °C for 20–22 h with 1 mM IPTG. The cultures were centrifuged at 4,400 rpm and 4 °C for 30 min. Cell pellets were resuspended in lysis buffer (25 mM Tris-HCl pH 7.5, 25 mM NaCl, 1 mM β-ME, 10% glycerol, 1:1,000 lysozyme, aprotinin/leupeptin, Pefabloc®), homogenized via sonication and centrifuged at 4 °C and 18,000 rpm for 30 min. The cleared supernatants were purified via Ni-NTA affinity chromatography (HisTrap crude FF column, Cytiva) with elution buffer (50 mM Tris-HCl pH 8.0, 0.5 M NaCl, 1 mM MgCl2, 1 mM βME, 0.5 M imidazole). Selected fractions containing the desired proteins were dialyzed and digested by TEV_{opt} protease[1] (protein/TEV 1:50 w/w) against TEV-buffer (20 mM Tris-HCl pH 8.0, 0.1 M NaCl, 1 mM $MgCl_2$, 1 mM βME) at 4 °C overnight in a dialysis tubing (SnakeSkin 30K MWCO, Thermo Fisher Scientific). The dialyzed proteins were then purified via anion exchange chromatography (HiTrap Q HP column, Cytiva) with elution buffer (20 mM Tris-HCl pH 8.0, 1 M NaCl). The purified proteins were dialyzed in storage buffer (20 mM Tris-HCl pH 7.4, 0.1 M NaCl, 1 mM $MgCl_2$, 1 mM TCEP), subsequently supplemented with 10% glycerol and concentrated to a concentration of approx. 8 mg/mL for a storage at −20 °C or −80 °C.
For BiP T229A expression, His_6-TEV fusion protein of BiP T229A (aa 19-654) coded on a pProEX vector was expressed in *E. coli* BL21 (DE3) in LB medium containing 100 µg/mL to a density of A600 = 0.6–0.7. Protein expression was induced at 23 °C for 20–22 h with 1 mM IPTG. The culture was centrifuged at 4,400 rpm and 4 °C for 30 min. Cell pellet was resuspended in lysis buffer (25 mM Tris-HCl pH 7.5, 25 mM NaCl, 1 mM β-ME, 10% glycerol, 1:1,000 lysozyme, aprotinin/leupeptin, Pefabloc®), homogenized via sonication and centrifuged at 4 °C and 18,000 rpm for 30 min. The cleared supernatant was purified via Ni-NTA affinity chromatography (HisTrap crude FF column, Cytiva) with elution buffer (50 mM HEPES pH 7.5, 0.4 M NaCl, 0.5 M imidazole). Selected fractions containing the desired protein were dialyzed and digested by TEV_{opt} protease (protein/TEV 1:50 w/w) against TEV-buffer at 4 °C overnight in a dialysis tubing (SnakeSkin 30K MWCO, Thermo Fisher Scientific). Then, another Ni-NTA affinity chromatography with the same conditions was performed. The flow through was collected and dialyzed against size-exclusion buffer (50 mM HEPES pH 7.5, 150 mM KCl, 10 mM MgCl2). The dialyzed protein was subjected to size-exclusion chromatography (HiLoad 16/600, 200 pg column, Cytiva) using size exclusion buffer. The purified protein was dialyzed in storage buffer (40 mM HEPES pH 7.4, 120 mM KCl, 8 mM $MgCl_2$) and concentrated to a concentration of approx. 18 mg/mL for a storage at −20 °C or −80 °C.
Protein concentrations were determined by BCA assay kit (Thermo Fisher Scientific) and purity was confirmed by LC-HRMS.

***In vitro* AMPylation assays.**
AMPylation assays were generally performed in AMPylation buffer (20 mM Tris-HCl pH 7.4, 0.1 M NaCl, 1 mM $MgCl_2$, 1 mM TCEP) containing the respective nucleotide and either wt FICD (aa 102-458) or FICD E234G (aa 102-458).
For autoAMPylation assays, reaction mixtures of 0.5 µM enzyme, the respective amount of nucleotide and 68 µg/mL BSA as loading control in AMPylation buffer were prepared. The mixtures were incubated at 37 °C for 1 h, subsequently quenched with 1x SDS-PAGE loading buffer and boiled at 95 °C for 5 min. The assays were then resolved by SDS-PAGE and analyzed via Western blot.
For substrate AMPylation, recombinant BiP T229A, Bub3 and EIF3D were tested. Bub3 (aa 1-328, His-tag) and EIF3D (aa 1-548, C-Myc/DDK tag) were purchased from OriGene. Reaction mixtures of 0.25 µM enzyme and 2.5 µM substrate with the respective nucleotide in AMPylation buffer were prepared. If shown on the blot, HA-tagged Hdm2 (HA-Hdm2) protein was added as loading control. The mixtures were incubated at 37 °C for 1 h, subsequently quenched with 1x SDS-PAGE loading buffer and boiled at 95 °C for 5 min. The assays were then resolved by SDS-PAGE and analyzed via Western blot.
For full-length protein MS analysis, reaction mixtures of 1 µM of FICD E234G, 100 µM nucleotide in AMPylation buffer were prepared. The mixtures were incubated at 37 °C for 1 h and subsequently reactions were quenched with 10 mM EDTA at 0 °C. The samples were concentrated to achieve a concentration of 7.5 µM of FICD E234G. The samples were then analyzed by LC-MS.

For peptide MS/MS analysis, AMPylation reactions were prepared as for autoAMPylation or substrate AMPylation except for the loading control. Samples were resolved by SDS-PAGE and then prepared for LC-MS/MS analysis.

Western blot.
Reaction samples for Western blot analysis were first resolved via SDS-PAGE. Afterwards, the proteins were transferred to PVDF membrane (0.2 µm, GE Healthcare) via electrophoresis in transfer buffer (12.5 mM Tris-HCl pH 8.3, 0.1 M glycine). The membrane was blocked with Roti®-Block (Carl Roth) in TNE-T buffer (10 mM Tris-HCl pH 7.6, 50 mM NaCl, 2.5 mM EDTA and 0.1% Tween® 20) at r.t. for 1 h. When BSA or HA-Hdm2 were blotted on the membrane, the membrane was cut on the 55 kDa or 100 kDa marker, respectively, and incubated with the following primary antibodies at 4 °C overnight. The lower membrane part was decorated with α-AMP-antibody 17G633 (1:1,000 in 1x TNE-T, mouse, monoclonal), the upper part with α-BSA (1:1,000 in 1x TNE-T, mouse monoclonal, Santa Cruz Biotechnology) or α-HA.11 antibody (1:2,000 in 1x TNE-T, mouse monoclonal, BioLegend), respectively. After washing with TNE-T buffer, HRP-coupled α-mouse antibody (1:30,000 in 1x TNE T, goat, Dianova) was used as secondary antibody at room temperature for 1 h. Finally, the blot was again washed with TNE-T buffer and visualized with the help of Western Bright ECL kit (advansta) on an Amersham Imager 600.

Cell culture and lysis.
NUDT2-deficient HeLa cells were grown in Dulbecco's Modified Eagle Medium (DMEM, Gibco) supplemented with 10% (v/v) fetal calf serum (Gibco) and 100 units/mL penicillin/streptomycin at 37 °C and 5% vol. CO2. Cells were harvested by scratching in 1x PBS on ice and centrifuged at 800 g and 4 °C for 15 min. Cells were washed with 1x PBS and centrifugation was repeated. The supernatant was discarded and the pellets were stored at −80 °C until usage. For lysis, cells were resuspended in lysis buffer (1x PBS, 1 mM EDTA, cOmplete protease inhibitor cocktail, Roche), sonicated and the lysates were cleared by centrifugation at 14,000 g and 4 °C for 20 min. The protein content of the supernatant was determined by BCA assay kit (Thermo Fisher Scientific).

Fluorescence analysis.
For analysis of cell lysate via fluorescence read-out, mixtures of 2 mg/mL HeLa KO cell lysate, 1 µM wt FICD and 200 µM *N*6-pATP or *N*6-pAp4A in AMPylation buffer were prepared and incubated at 37 °C for 1 h. Then, MeOH was added four times the volume of AMPylation mixture to quench the reaction and the solution was incubated at −20 °C for 2 h. The samples were centrifuged at 14,000 g and 4 °C for 20 min. The supernatant was discarded and the pellet was dissolved in 4ST buffer (4% SDS, 150 mM NaCl, 50 mM TEA pH 7.4). For CuAAC, 0.1 mM TBTA, 1 mM $CuSO_4$, 1 mM TCEP and 0.2 mM N_3-Sulfo-Cy5 were added to each sample. Subsequently, reaction was quenched with 1x SDS-PAGE loading buffer and boiled at 95 °C for 5 min. The samples were then resolved by SDS-PAGE and fluorescence read-out was performed on a Typhoon FLA 9500 (λ_{ex} = 635 nm).

Affinity purification assay.
For protein enrichment, AMPylation assays were first prepared. Mixtures of 2 mg/mL HeLa KO cell lysate, 1 µM wt FICD and 200 µM *N*6-pATP or *N*6-pAp4A in AMPylation buffer were prepared and incubated at 37 °C for 1 h. Then, MeOH was added four times the volume of AMPylation mixture to quench the reaction and the solution was incubated at −20 °C for 2 h. The samples were centrifuged at 14,000 g and 4 °C for 20 min. The supernatant was discarded and the pellet was dissolved in 4ST buffer (4% SDS, 150 mM NaCl, 50 mM TEA pH 7.4). For CuAAC, 0.1 mM TBTA, 1 mM CuSO4, 1 mM TCEP and 0.2 mM N_3-(Arg)-PEG_3-DB were added to each sample. The samples were incubated at 25 °C for 1 h and subsequently acetone was added four times the volume of the samples. Upon overnight incubation at −20 °C, samples were centrifuged at 14,000 g and 4 °C for 10 min. The supernatant was removed and the pellet was washed with MeOH three times. The dried pellet was dissolved in 0.8% SDS in 1x PBS and incubated with Pierce™ Streptavidin Agarose (Thermo Fisher Scientific) at 30 °C for 30 min. The beads were washed with 1% SDS in 1x PBS (3x), 1x PBS containing 1% SDS, 6 M urea and 150 mM NaCl (6x), and 50 mM NH_4HCO_3 (5x). Afterwards, elution was realized with 0.8 mM biotin and 0.1% RapiGest SF tenside (Waters Corporation) in 50 mM NH_4HCO_3 at 37 °C for 10 min. Elution was performed three times. Eluted samples were then treated for in-solution digestion. Samples were incubated with 5 mM DTT at 60 °C for 30 min, cooled to r.t. and alkylated by addition of 50 mM CAA at r.t. for 30 min. Next, proteins were digested by trypsin (1:50 w/w, Promega) overnight at 37 °C. Digestion was quenched with 0.5 % TFA, the samples were centrifuged at 14,000 g and r.t. for 15 min and finally freeze-dried. Freeze-dried samples were desalted using uC18 Zip Tips (Merck) before LC-MS/MS measurement.

LC-MS/MS measurement of affinity purification samples.
Samples were analyzed on a Q-Exactive HF Mass Spectrometer (Thermo Fisher Scientific) coupled to an EASY nLC 1200 UHPLC System (Thermo Fisher Scientific) equipped with a 50 µm x 15 cm C18 Acclaim PepMap RSLC column with 2 µm beads and 100 Å pore size (Thermo Fisher Scientific). Peptides were eluted in a 90 min gradient at a flow rate of 300 nL/min starting from 5% ACN, 0.1% FA, to 30% ACN, 0.1% FA in 75 min followed by a washing step of 10 min at 80% ACN, 0.1% FA. Samples were measured in technical duplicates with a data-dependent acquisition method. MS1 survey scans from 350 to 1,400 m/z were acquired at a resolution of 120,000, an AGC target of 3e6 and a maximum injection time of 60 ms followed by isolation and HCD fragmentation of no more than 15 of the

most intense precursor ions with a normalized collision energy of 28 eV. Fragment spectra were acquired at a resolution of 15,000, an AGC target of 1e5 and with a maximum injection time of 50 ms. The intensity threshold was set to 2e4 and dynamic exclusion was set to 30 s.

Data analysis and quantification of affinity purification assays.
Raw files from LC-MS/MS measurements were analyzed using MaxQuant (version 2.0.3.0) with the andromeda search engine with default settings and match between runs and LFQ (minimum ratio count 2) enabled[2,3]. For protein identification, the human reference proteome downloaded from the UniProt database (download date: 02.04.2019, https://www.uniprot.org/proteomes/) and an integrated database of common contaminants were used. Oxidation and carbamidomethylation were selected as modifications. Identified proteins were filtered for hits only identified by site, reverse hits and potential contaminants at a false discovery rate (FDR) of ≤ 0.01. Raw files of the affinity purification assay described above were analyzed by MaxQuant together with files from an affinity purification assay with *C*2-ethynyl-modified ATP or Ap_4A with and without addition of wt FICD as well as *N*6-pATP and *N*6-pAp_4A without addition of wt FICD and an control experiment with addition of shrimp alkaline phosphatase to all modified Ap_4A assays (all measurements uploaded on PRIDE, dataset identifier PXD036450). During further data processing with Perseus software (version 1.6.14.0)[4], all measurements except for the assay with *N*6-pATP, *N*6-pAp_4A and addition of wt FICD including a bead control were discarded. This was due to potential side reactions of *C*2-ethynyl-modified nucleotides (not discussed in this work) and low labeling efficiency in assays without addition of wt FICD (data not shown). LFQ intensities were $\log_2$ transformed and proteins were filtered to be quantified in at least 4 out of the 6 replicates of one condition (bead control, *N*6-pATP or *N*6-pAp_4A). Missing values were imputed from a normal distribution (width = 0.3 and shift = 1.8) separately for each column. Enriched proteins were identified by a one-way ANOVA-based multiple sample test with S_0 adjusted to 0.3 and the permutation-based FDR accepted ≤ 0.02 with technical replicates grouped for randomization. Enriched proteins were grouped by mean and *Z*-scoring of the median LFQ intensities was performed. Analysis of the enriched proteins was performed by hierarchical clustering of all significant hits. Euclidean distance was used, the linkage was set to average and the maximal numbers of clusters to 300. Proteins with a *Z*-score difference of *N*6-pAp_4A and *N*6-pATP of ≥ 0.75 were considered significantly enriched for *N*6-pAp_4A and thus considered for further analysis.
Functional classification of *N*6-pAp_4A-enriched proteins with respect to their GO terms was realized by Protein ANalysis THrough Evolutionary Relationships (PANTHER) database (version 17.0, Supplementary data 2)[5] regarding biological process and molecular function. GO annotation was also examined via Database for Annotation, Visualization and Integrated Discovery (DAVID, version 6.18)[6,7] regarding cellular component and molecular function (supplementary data 3).

In gel digestion and sample preparation.
A modified protocol from Shevchenko et al.[8] was used. After resolution of the samples via SDS-PAGE followed by Coomassie staining, protein bands were excised from the gel and destained in destain buffer (50 mM NH_4H/ACN, 1:1, v/v) at 37 °C. The gel pieces were incubated with reduction buffer (10 mM DTT in 50 mM NH_4HCO_3) at 56 °C for 1 h and subsequently in alkylation buffer (50 mM chloroacetamide in 50 mM NH_4HCO_3) at r.t. for 1 h. After washing with destain buffer, the pieces were dried in ACN followed by trypsin digestion at 37 °C overnight. The supernatant was stored and further peptide extraction from the gel was performed. First, the pieces were incubated in 5% FA/ACN solution (2:1, v/v) at 37 °C for 15 min, then sonicated for 15 min and the supernatant was removed. This was repeated with 5% FA/ACN solution (1:2, v/v). All extracts were combined and freeze-dried. Freeze-dried samples were desalted using uC18 Zip Tips (Merck) before LC-MS/MS measurement and analysis.

LC-MS/MS measurement of in gel digested proteins.
Samples were analyzed on the same LC-MS system described for affinity purification samples. Peptides were eluted in a 45 min gradient at a flow rate of 300 nL/min starting from 2.5% ACN, 0.1% FA, to 25% ACN, 0.1% FA in 30 min, to 32% ACN, 0.1% FA in 5 min followed by a washing step of 5 min at 75% ACN, 0.1% FA. Samples were measured in technical duplicates with a data-dependent acquisition method. MS1 survey scans from 350 to 1,600 m/z were acquired at a resolution of 120,000, an AGC target of 3e6 and a maximum injection time of 60 ms followed by isolation and HCD fragmentation of no more than 15 of the most intense precursor ions with a normalized collision energy of 28 eV. Fragment spectra were acquired at a resolution of 30,000, an AGC target of 1e5 and with a maximum injection time of 120 ms. The intensity threshold was set to 2e4. Precursor ions that had been selected for fragmentation were excluded from isolation for 5 s.

Identification of AMPylated peptide fragments.
Raw data files were converted to Mascot generic format using ProteoWizard and subjected to a database search against the amino acid sequence of FICD wt or E234G, BiP or Bub3 using Mascot (version 2.5.1) as search engine. Carbamidomethylation of cysteine was set as fixed modification and oxidation of methionine as well as AMPylation of serine/threonine/tyrosine (Delta mass of 329.053 Da) were set as variable modifications. For AMPylation, neutral losses according to the loss of one or more of the three characteristic reporter ions during MS acquisition were included.[9,10] Reporter ion intensities of the three reporter ions (136.0623 Da, 250.0940 Da and 348.0709 Da) were quantified with a Mascot reporter ion quantification method at a tolerance of 10 ppm. Peptide ion tolerance for the database search was set to 10 ppm, fragment ion tolerance was set to 0.02 Da. A maximum of two missed tryptic cleavages was

allowed. Putative peptide spectrum matches of AMPylated peptides as indicated from the Mascot database search were additionally filtered for the presence of at least two AMPylation reporter ions[9,10].

LC-MS measurement and analysis of full-length proteins.

For LC-MS measurement of full-length proteins, samples were separated on an analytical ProSwift™ RP-4H (Analytic, 1 X 250mm; Thermo Fisher Scientific) connected to a liquid chromatography system (Agilent, Infinity II 1260). Samples were resolved at a flow rate of 300 µL/min. A gradient of 5–25% solvent B (100% ACN) over 20 min was used, solvent A (0.1% FA in MQ). Mass spectra were collected on a 6546 QTOF mass spectrometer (Agilent) equipped with a dual AJS ESI source and operated in positive mode with an acquisition rate of 1,000 ms/spectra, 320 °C gas temperature, 8 L/min drying gas, 35 psi nebulizer, 350 °C sheath gas temperature and 11 L/min sheath gas flow. Fragmentor was set to 175 V, skimmer to 65 V, Vcap to 3,500 V and Nozzle Voltage was set to 1,000 V. Data were recorded by MassHunter (version 10.1.62, Agilent) Peaks were deconvoluted by MassHunter BioConfirm (version 10.0.10136.0, Agilent) with settings for maximum entropy. Mass steps were set 0.05 Da, limited m/z range was set to 600.00-2500.00 m/z and mass range was set to 35,000-50,000 Da. Baseline was subtracted by the factor 7.0 and searched adducts were protonated signals with peak signals to noise ≥30.0 counts, minimum charge state 5 and minimum protein fit score 8. Spectra were deconvoluted within a specific retention time range set to 15.5-17.5 min.

MS data deposition

All mass spectrometry data have been deposited to the ProteomeXchange Consortium via the PRIDE[11] partner repository with the dataset identifier PXD036450 (Username: reviewer_pxd036450@ebi.ac.uk, Password: SAkmw1It).

Chemical synthesis of of *N*6, *N* 6''-dipropargyldiadenosine-*O*5',*O*5'''-tetraphosphate (sodium salt form, 3)

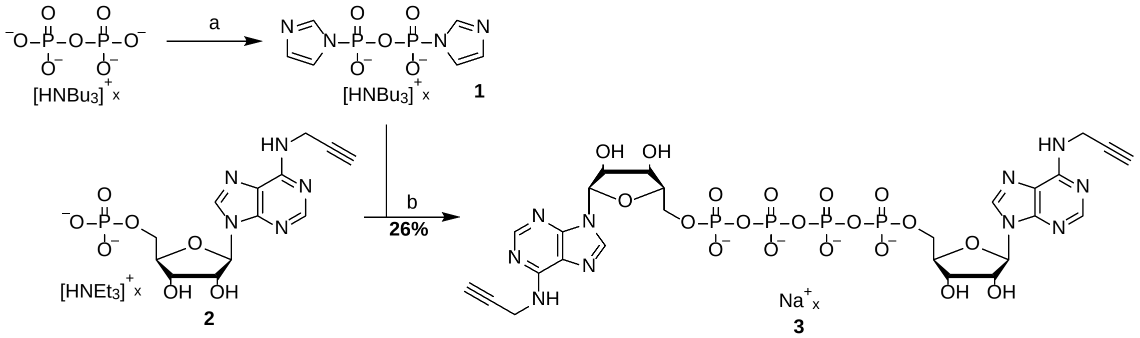

Scheme S1. Synthesis of *N*6,*N*6''-dipropargyldiadenosine-*O*5',*O*5'''-tetraphosphate (*N*6-pAp4A, **3**). Conditions: a) carbonyldiimidazole, NEt_3, r.t., 15 min. b) 0.45 M tetrazole in ACN, DMF, r.t., o.n.

The synthesis of *N*6,*N*6''-dipropargyldiadenosine-*O*5',*O*5'''-tetraphosphate (*N*6-pAp_4A) was performed by following the procedure published by Yanachkov *et al.*[12]. *N*6-propargyladenosine-5'-phosphate (**2**) was prepared as previously published[13]. Pyrophosphate tributylammonium salt (57 µmol, 31 mg, 0.5 eq) and carbonyldiimidazole (226 µmol, 28 mg, 1.5 eq) were suspended in dry DMF (2 mL). Triethylamine (57 µmol, 8 µL, 0.5 eq) was added and the reaction was stirred at r.t. for 15 min. H_2O (15 µL) was added and the solvent was removed. The residue containing compound 1 was dissolved in dry DMF and added to *N*6-propargyladenosine-5'-phosphate triethylammonium salt (**2**, 113 µmol, 1.0 eq) in dry DMF (2 mL). Then, tetrazole (0.45 M in ACN, 102 µmol, 226 µL, 0.9 eq) was added. The reaction was stirred at r.t. overnight and subsequently quenched with 0.1 M TEAB (pH 7.5). The solvents were evaporated and dissolved in MQ water. The mixture was purified via anion-exchange chromatography (DEAE Sephadex™ A-25) and RP-HPLC, successively. Fractions containing the product were concentrated under reduced pressure and repeatedly freeze-dried. The resulting powder was dissolved in MQ water and added to $NaClO_4$ in acetone (0.5 g in 20 mL). The resulting precipitate was centrifuged at 4,400 rpm for 10 min and the supernatant was removed. The residue was again dissolved in MQ water and addition to $NaClO_4$ in acetone and centrifugation was repeated. The pellet was washed with pure acetone and dried in vacuo. *N*6-pAp_4A as sodium salt (14.7 µmol, 26%) was obtained as a white powder.

^{1}H NMR (800 MHz, D_2O) δ=8.32 (s, 2H, H-8, H-8''), 8.20 (s, 2H, H-2, H-2''), 6.02 (d, *J*=5.7 Hz, 2H, H-1', H-1'''), 4.73 (t, *J*=5.4 Hz, 2H, H-2', H-2'''), 4.56 (t, *J*=4.4 Hz, 2H, H-3', H-3'''), 4.35 (m, 2H, H-4', H-4'''), 4.29 (m, 1H, H_a-5', H_a-5'''), 4.23 (m, 6H, H_b-5', H_b-5''', *N*6-C*H₂*C≡CH, N6''-C*H₂*C≡CH), 2.63 (s, 2H, *N*6-CH2C≡C*H*, *N*6''-CH2C≡C*H*) ppm. ^{13}C NMR (201 MHz, D2O) δ=153.3 (C-6, C-6''), 152.4 (C-2, C-2''), 148.0 (C-4, C-4''), 139.4 (C-8, C-8''), 118.5 (C-5, C-5''), 86.6 (C-1', C-1'''), 83.7 (d, J = 9.1 Hz, C-4', C-4'''), 79.9 (d, *J*=91.8 Hz, *N*6-CH_2*C*≡CH, N6''-CH_2*C*≡CH), 74.5 (C-2', C-2'''), 71.9 (*N*6-CH_2C≡*C*H, *N*6''-CH_2C≡*C*H), 70.3 (C-3', C-3'''), 65.1 (d, *J*=4.2 Hz, C-5', C-5'''), 30.0 (*N*6-*C*H_2C≡CH, *N*6''-*C*H_2C≡CH) ppm. ^{31}P NMR (324 MHz, D_2O) δ=−11.2 (m, 1P, Pα, Pα'), −22.9 (m, 1P,

Pβ, Pβ') ppm. HRMS (ESI): *m/z* calcd for $C_{26}H_{31}N_{10}O_{19}P_4^-$: 911.0723 [*M*−H]$^-$; found: 911.0726; calcd for $C_{26}H_{30}N_{10}O_{19}P_4^{2-}$: 455.0325 [*M*−H]$^{2-}$; found: 455.0324.

Supporting Results

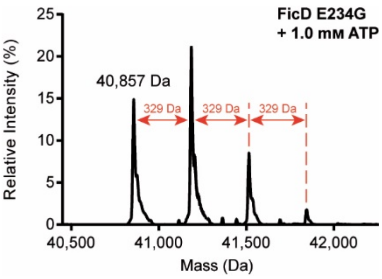

Figure S1. Intact protein LC-MS analysis of FICD E234G incubated with 1 mM ATP. Spectrum pattern is similar as for 100 µM ATP.

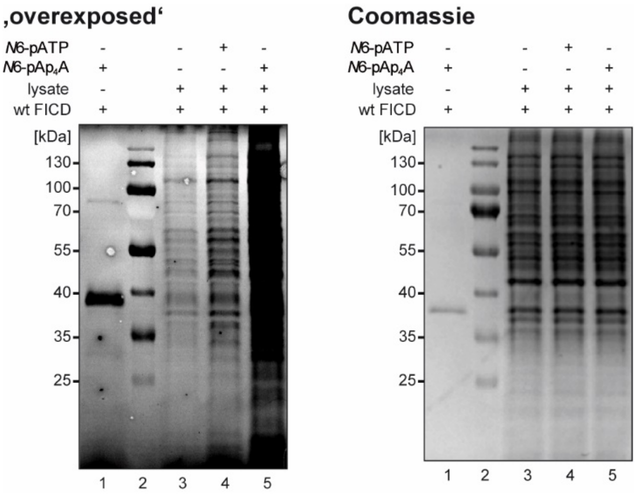

Figure S2. Additional analysis of the fluorescence read-out of HeLa KO lysate incubated with chemical reporters and wt FICD (as shown in Figure 2c). Left: 'overexposed' image (altered black/white balance) of the fluorescence read-out for visualization of *N*6-pATP labeled bands. Right: Coomassie blue stained gel of the experiment serving as loading control.

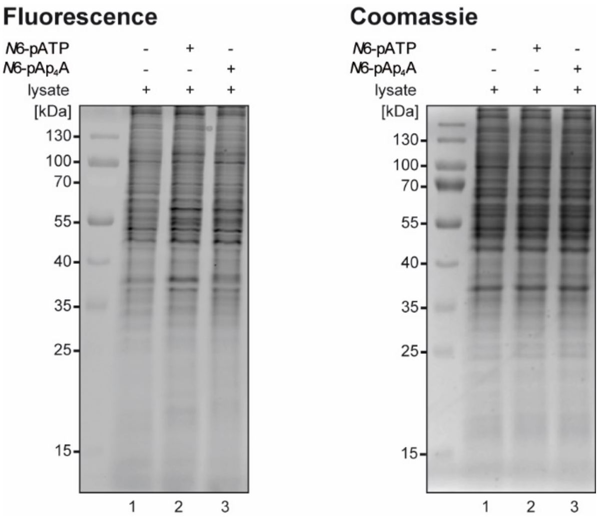

Figure S3. Analysis of the fluorescence read-out of HeLa KO lysate incubated with chemical reporters but without wt FICD. Left: Fluorescence read-out upon labelling with N_3-Sulfo-Cy5. Right: Coomassie blue stained gel of the experiment serving as loading control.

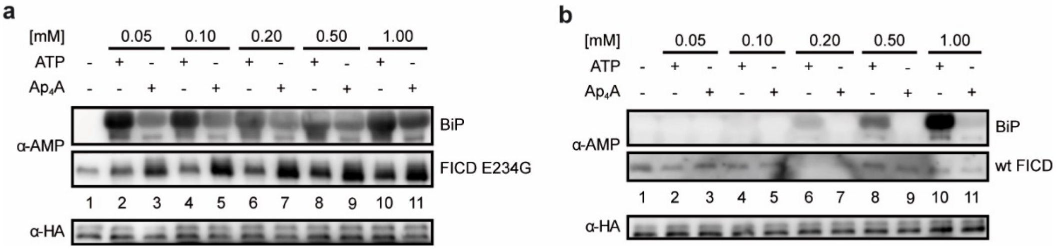

Figure S4. Target AMPylation of BiP. **a)** Western blot of the concentration dependent AMPylation of BiP mediated by FICD E234G using natural ATP or Ap$_4$A as cosubstrate spiked with ApCpp (1:10 ApCpp/natural nucleotide concentration) serving as surrogate for BiP ATP binding. Detection of AMPylation was realized via α-AMP-antibody and HA serving as loading control against α-HA-antibody. **b)** Same as a but with wt FICD.

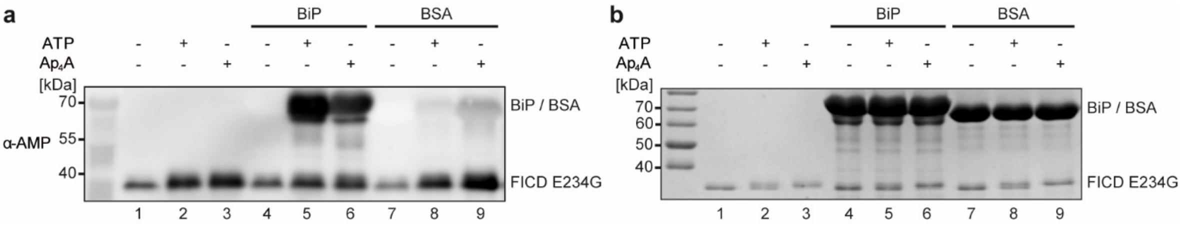

Figure S5. Specificity test of target AMPylation. **a)** Western blot of autoAMPylation of FICD E234G (lanes 1-3), target AMPylation of BiP (lanes 4-6) and BSA (as negative control, lanes 7-9) mediated by FICD E234G using natural ATP or Ap$_4$A as cosubstrate. Detection of AMPylation was realized via α-AMP-antibody. **b)** Coomassie blue stained SDS-PA gel of the experiment serving as control.

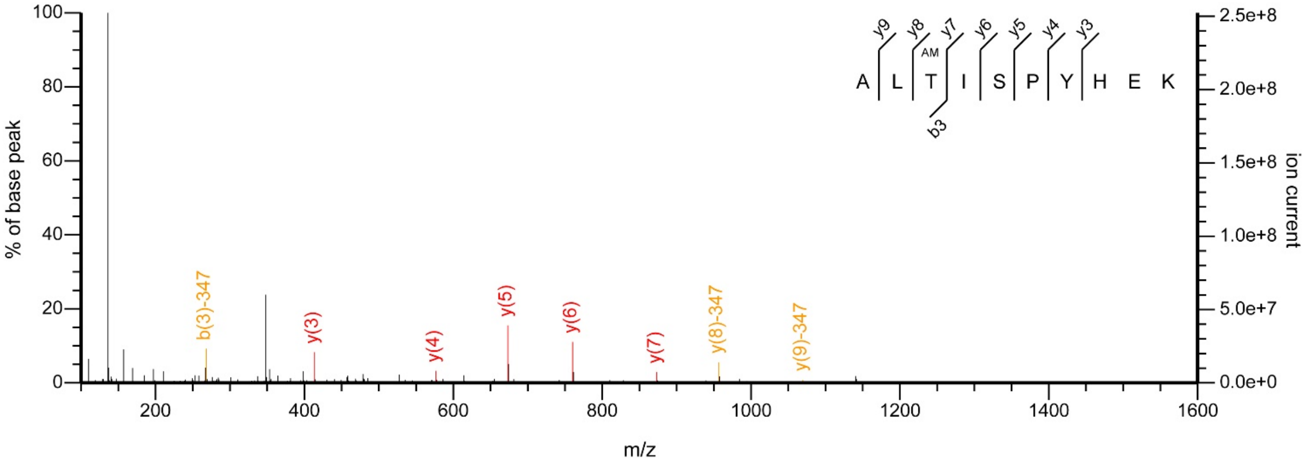

Figure S6. MS/MS spectrum of AMPylated peptide (single peptide ID) containing Thr168 of FICD E234G using Ap$_4$A as cosubstrate. MS/MS spectrum for the peptide was selected based on the highest score (-10lgP) assigned by Mascot. Fragment ions with a characteristic AMPylation associated neutral loss are shown in yellow.

ATP

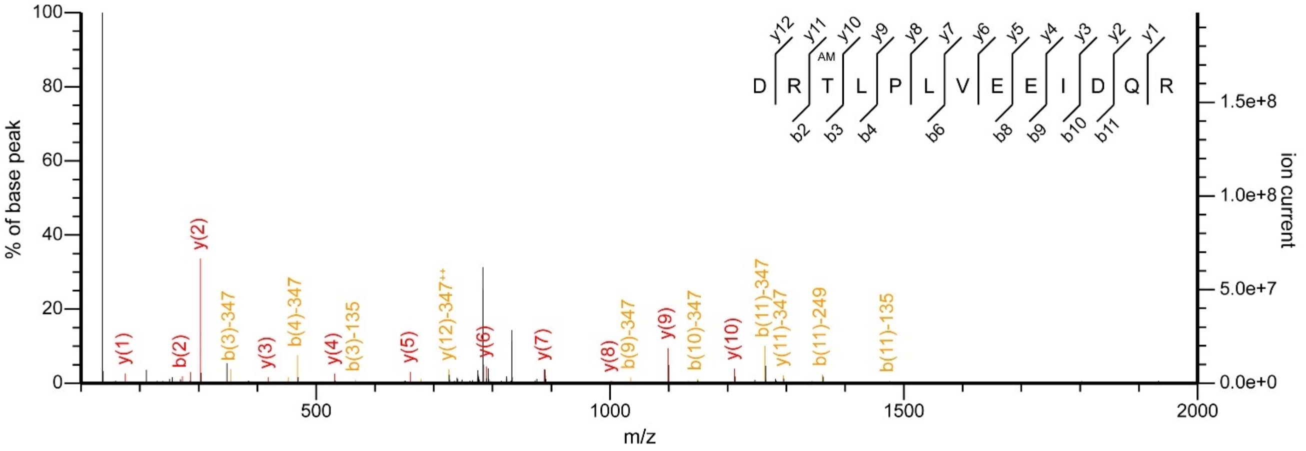

Ap$_4$A

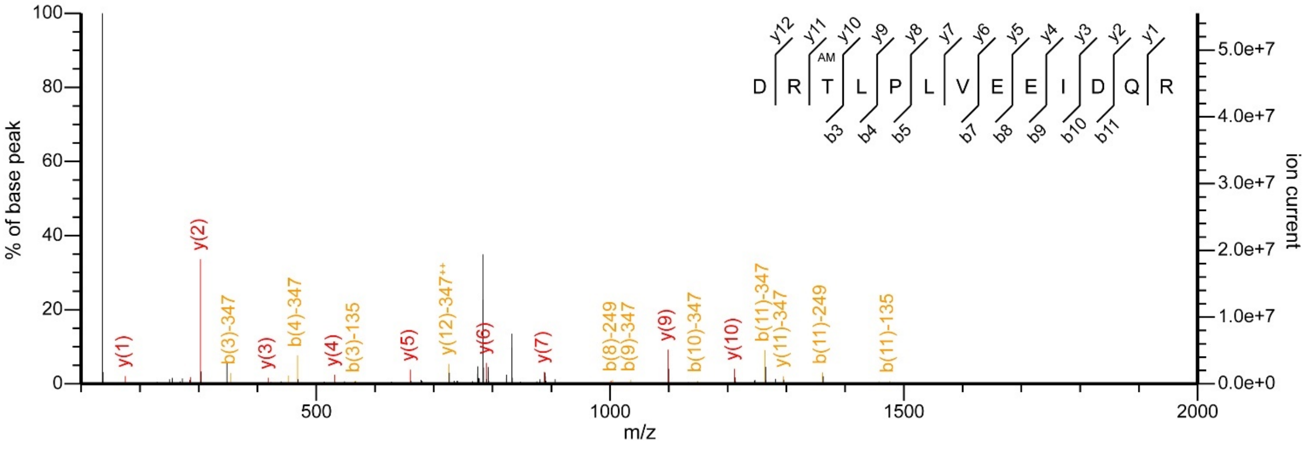

Figure S7. MS/MS spectrum of AMPylated peptide (single peptide ID) containing Thr183 of FICD E234G using ATP (top) or Ap$_4$A (bottom) as cosubstrate. MS/MS spectrum for the peptide was selected based on the highest score (-10lgP) assigned by Mascot. Fragment ions with a characteristic AMPylation associated neutral loss are shown in yellow.

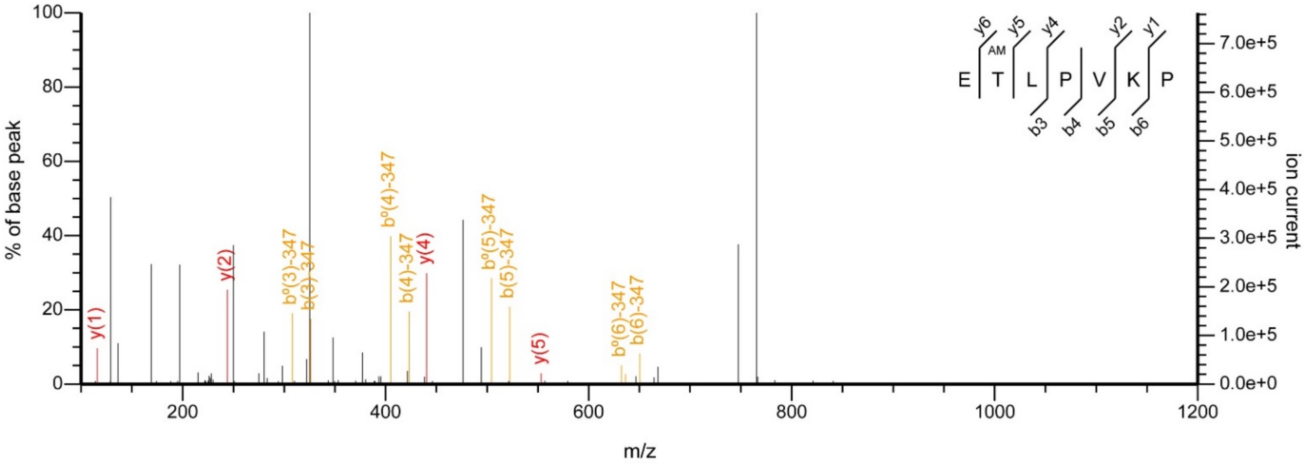

Figure S8. MS/MS spectrum of AMPylated peptide (single peptide ID) containing Thr453 of FICD E234G using Ap$_4$A as cosubstrate. MS/MS spectrum for the peptide was selected based on the highest score (-10lgP) assigned by Mascot. Fragment ions with a characteristic AMPylation associated neutral loss are shown in yellow.

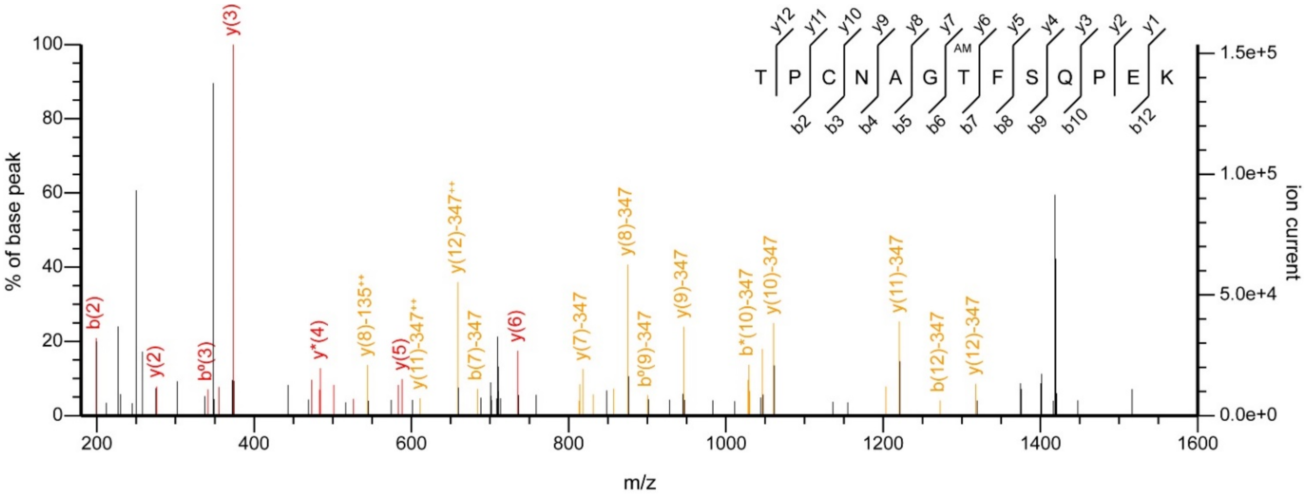

Figure S9. MS/MS spectrum of AMPylated peptide (single peptide ID) containing Thr518 of BiP T229A using Ap_4A as cosubstrate. MS/MS spectrum for the peptide was selected based on the highest score (-10lgP) assigned by Mascot. Fragment ions with a characteristic AMPylation associated neutral loss are shown in yellow.

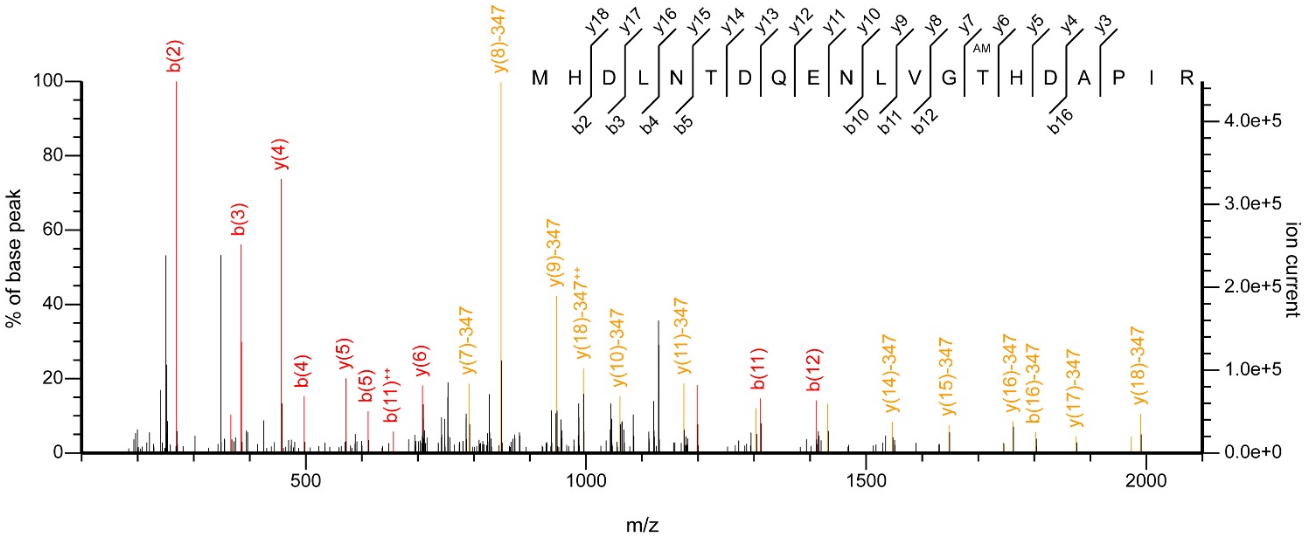

Figure S10. MS/MS spectrum of AMPylated peptide (single peptide ID) containing Thr94 of Bub3 using ATP as cosubstrate. MS/MS spectrum for the peptide was selected based on the highest score (-10lgP) assigned by Mascot. Fragment ions with a characteristic AMPylation associated neutral loss are shown in yellow.

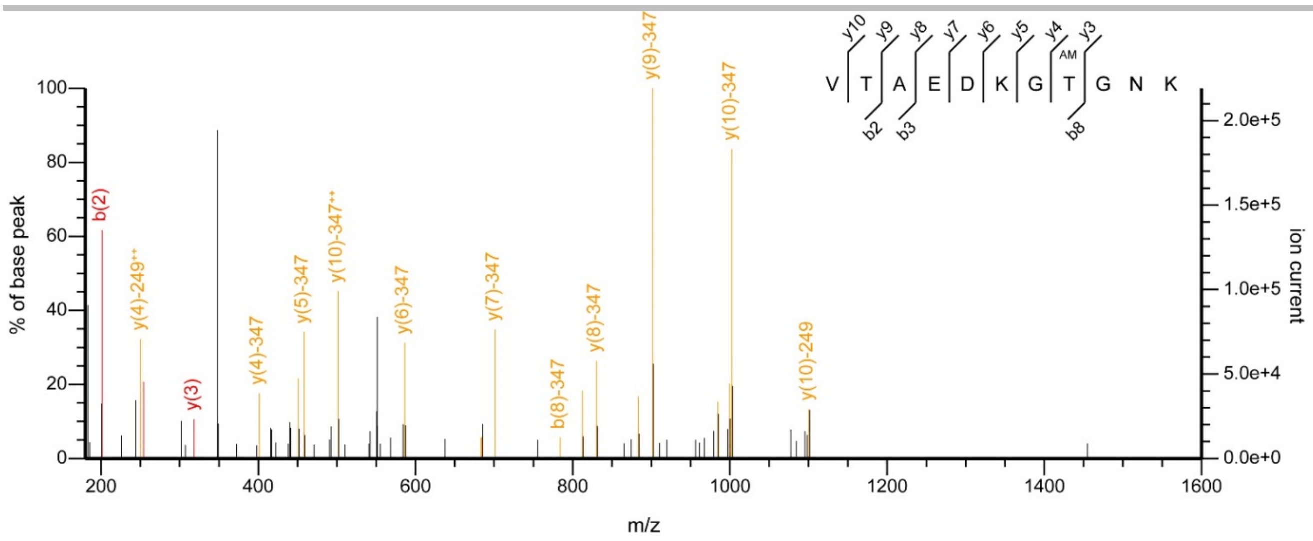

Figure S11. MS/MS spectrum of AMPylated peptide (single peptide ID) containing Thr133 of Bub3 using Ap4A as cosubstrate. MS/MS spectrum for the peptide was selected based on the highest score (-10lgP) assigned by Mascot. Fragment ions with a characteristic AMPylation associated neutral loss are shown in yellow.

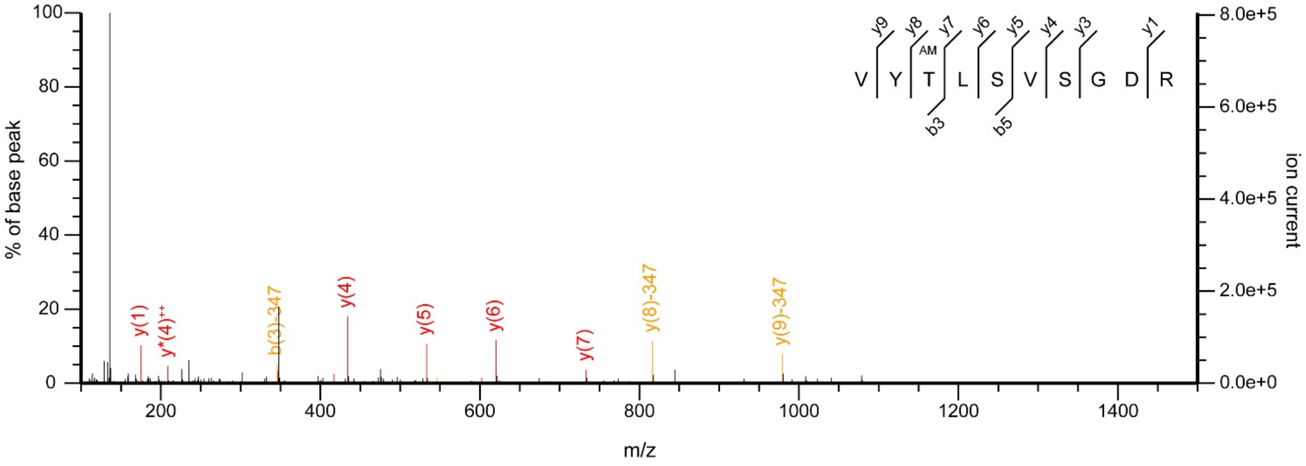

Figure S12. MS/MS spectrum of AMPylated peptide (single peptide ID) containing Thr142 of Bub3 using Ap4A as cosubstrate. MS/MS spectrum for the peptide was selected based on the highest score (-10lgP) assigned by Mascot. Fragment ions with a characteristic AMPylation associated neutral loss are shown in yellow.

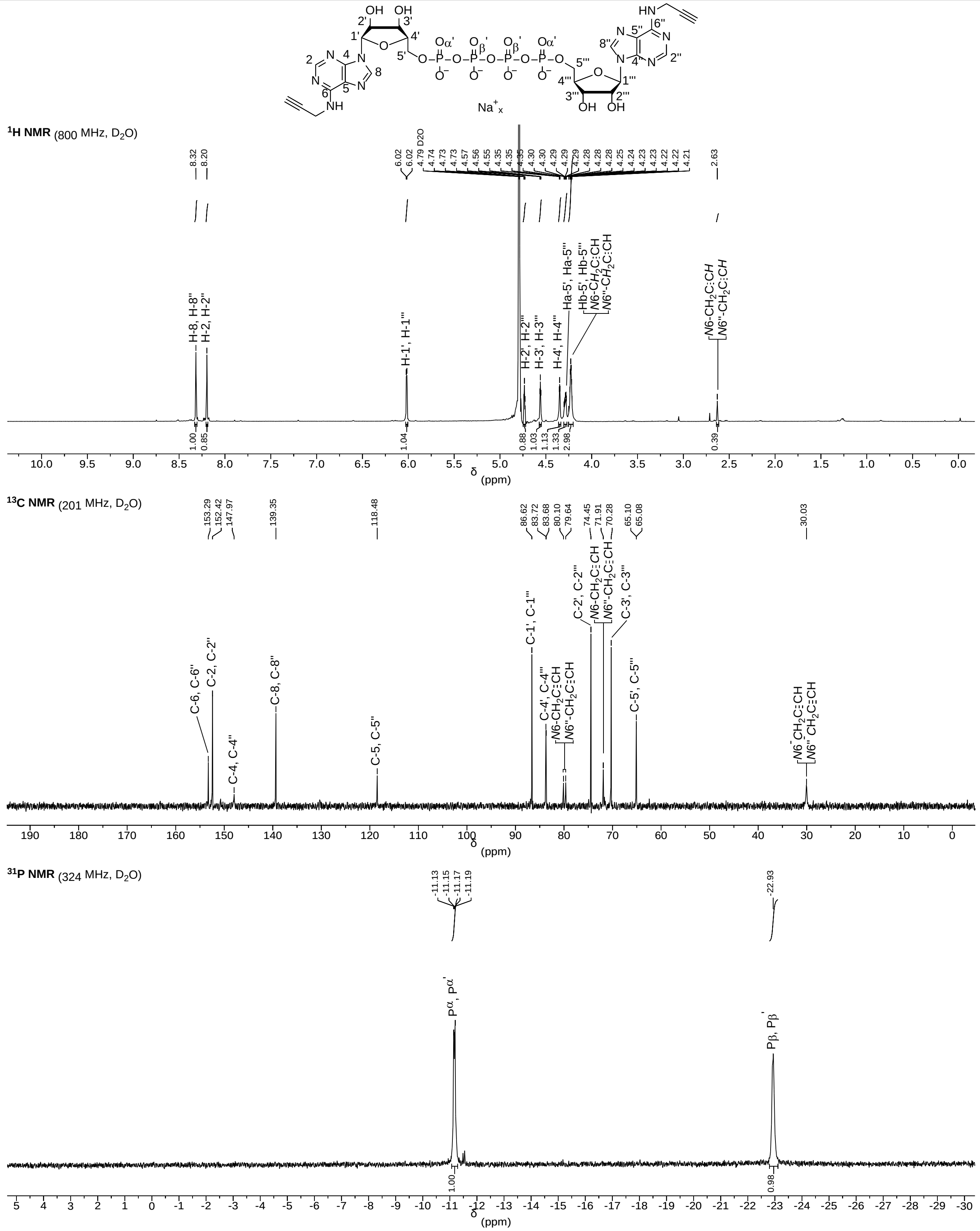

Figure S13. Structural characterization of *N*6,*N*6''-dipropargyldiadenosine-*O*5',*O*5'''-tetraphosphate (**3**) by nuclear magnetic resonance spectroscopy (NMR).

References

[1] S. Oesterle, T. M. Roberts, L. A. Widmer, H. Mustafa, S. Panke, S. Billerbeck, *BMC Biol* **2017**, *15*, 100.
[2] J. Cox, M. Mann, *Nat Biotechnol* **2008**, *26*, 1367–1372.
[3] J. Cox, M. Y. Hein, C. A. Luber, I. Paron, N. Nagaraj, M. Mann, *Molecular & Cellular Proteomics* **2014**, *13*, 2513–2526.
[4] S. Tyanova, T. Temu, P. Sinitcyn, A. Carlson, M. Y. Hein, T. Geiger, M. Mann, J. Cox, *Nat Methods* **2016**, *13*, 731–740.
[5] H. Mi, D. Ebert, A. Muruganujan, C. Mills, L.-P. Albou, T. Mushayamaha, P. D. Thomas, *Nucleic Acids Res* **2021**, *49*, D394–D403.
[6] D. W. Huang, B. T. Sherman, R. A. Lempicki, *Nat Protoc* **2009**, *4*, 44–57.
[7] B. T. Sherman, M. Hao, J. Qiu, X. Jiao, M. W. Baseler, H. C. Lane, T. Imamichi, W. Chang, *Nucleic Acids Res* **2022**, *50*, W216–W221.
[8] A. Shevchenko, H. Tomas, J. Havli, J. V Olsen, M. Mann, *Nat Protoc* **2006**, *1*, 2856–2860.
[9] T. Hansen, M. Albers, C. Hedberg, A. Sickmann, *Proteomics* **2013**, *13*, 955–963.
[10] Y. Li, R. Al-Eryani, M. L. Yarbrough, K. Orth, H. L. Ball, *J Am Soc Mass Spectrom* **2011**, *22*, 752–761.
[11] Y. Perez-Riverol, J. Bai, C. Bandla, D. García-Seisdedos, S. Hewapathirana, S. Kamatchinathan, D. J. Kundu, A. Prakash, A. Frericks-Zipper, M. Eisenacher, M. Walzer, S. Wang, A. Brazma, J. A. Vizcaíno, *Nucleic Acids Res* **2022**, *50*, D543–D552.
[12] I. B. Yanachkov, E. J. Dix, M. I. Yanachkova, G. E. Wright, *Org. Biomol. Chem.* **2011**, *9*, 730–738.
[13] H. Jiang, J. Congleton, Q. Liu, P. Merchant, F. Malavasi, H. C. Lee, Q. Hao, A. Yen, H. Lin, *J Am Chem Soc* **2009**, *131*, 1658–1659.

Author Contributions

M.F., P.S., and A.M. conceived the study and experimental approach; M.F. performed AMPylation experiments, the organic synthesis, and proteomics study, Y.Y. synthesized the modified azide, P.S. and D.He. conducted the MS experiments. Y.Y. and D.Hö. provided expert expertise in AMPylation studies. All authors analyzed the data. M.F. and A.M. wrote the manuscript with input from all other authors.
